# Neural populations within macaque early vestibular pathways are adapted to encode natural self-motion

**DOI:** 10.1371/journal.pbio.3002623

**Published:** 2024-04-30

**Authors:** Mohammad Mohammadi, Jerome Carriot, Isabelle Mackrous, Kathleen E. Cullen, Maurice J. Chacron

**Affiliations:** 1 Department of Biological and Biomedical Engineering, McGill University, Montreal, Canada; 2 Department of Physiology, McGill University, Montreal, Canada; 3 Department of Biomedical Engineering, Johns Hopkins University, Baltimore, Maryland, United States of America; 4 Department of Otolaryngology-Head and Neck Surgery, Johns Hopkins University School of Medicine, Baltimore, Maryland, United States of America; 5 Department of Neuroscience, Johns Hopkins University School of Medicine, Baltimore, Maryland, United States of America; 6 Kavli Neuroscience Discovery Institute, Johns Hopkins University, Baltimore, Maryland, United States of America; Universidad de Salamanca, SPAIN

## Abstract

How the activities of large neural populations are integrated in the brain to ensure accurate perception and behavior remains a central problem in systems neuroscience. Here, we investigated population coding of naturalistic self-motion by neurons within early vestibular pathways in rhesus macaques (*Macacca mulatta*). While vestibular neurons displayed similar dynamic tuning to self-motion, inspection of their spike trains revealed significant heterogeneity. Further analysis revealed that, during natural but not artificial stimulation, heterogeneity resulted primarily from variability across neurons as opposed to trial-to-trial variability. Interestingly, vestibular neurons displayed different correlation structures during naturalistic and artificial self-motion. Specifically, while correlations due to the stimulus (i.e., signal correlations) did not differ, correlations between the trial-to-trial variabilities of neural responses (i.e., noise correlations) were instead significantly positive during naturalistic but not artificial stimulation. Using computational modeling, we show that positive noise correlations during naturalistic stimulation benefits information transmission by heterogeneous vestibular neural populations. Taken together, our results provide evidence that neurons within early vestibular pathways are adapted to the statistics of natural self-motion stimuli at the population level. We suggest that similar adaptations will be found in other systems and species.

## Abstract 2

Information about self-motion is detected by the vestibular end organs but how neural populations represent natural self-motion stimuli is unclear. This study shows that, in macaques, neurons in early vestibular pathways are adapted to the statistics of natural self-motion stimuli at the population level.

## Introduction

How neural populations represent sensory input (i.e., population coding) remains a central problem in systems neuroscience [1–10]. For example, it is known that individual neurons within a population demonstrate differences in their spiking activities in response to a given stimulus (i.e., display response heterogeneity) [11–13]. Theoretical studies have shown that, in some circumstances, such heterogeneity benefits information transmission by increasing the coding range [14–24]. Furthermore, correlations between neural activities can either increase or decrease information transmission depending on their structure [4,8,9,25]. Additional complexity as to how correlations influence information transmission arises because these are highly plastic. Indeed, correlations can be regulated by multiple factors including attention [26,27], single neuron firing properties (e.g., firing rate, response nonlinearities) [28–30], and stimulus attributes (e.g., spatial extent, frequency content, intensity) [30–33] (see [34] for review).

The vestibular system plays a vital role in generating reflexive behaviors such as the vestibular spinal and ocular reflexes, as well as our perception of self-motion and spatial orientation. Information about self-motion is detected by the vestibular end organs from which peripheral afferents directly synapse onto neurons within the vestibular nuclei [35]. A subclass of neurons within the vestibular nuclei termed vestibular-only can easily be distinguished from other neural classes within the vestibular nuclei based on their lack of eye movement sensitivity. Eye movement sensitive neurons within the vestibular nuclei project to the abducens nucleus and mediate the vestibulo-ocular reflex (VOR) (see [36] for review) [37–39]. In contrast, vestibular-only neurons mediate other important functions including self-motion perception and the control of vestibulo-spinal reflexes as they project to both the thalamus [40] and the spinal cord [41,42].

To date, however, the neural representation of self-motion within the vestibular nuclei has primarily been studied by single-unit and, in one case, multi-unit recordings during artificial stimulation [43–46]. Specifically, vestibular-only neurons display high-pass tuning characteristics with neural sensitivity increasing with increasing frequency and large phase leads (i.e., the neural firing rate reaches its maximum before the stimulus; see [36] for review). In contrast, the statistics of self-motion stimuli experienced during typical everyday activities (i.e., natural self-motion stimuli) differ fundamentally from those of artificial stimuli [47,48] (see [49] for review). Recent studies have shown that early vestibular pathways optimally encode natural self-motion stimuli (afferents: [50]; vestibular nuclei: [44,45]; vestibular thalamocortical neurons: [51]) (see [49,52] for review). Notably, because of both their tuning properties and trial-to-trial variability, single vestibular-only neurons can optimally encode natural self-motion stimuli through temporal whitening [44,45]. However, these studies were based on single-unit recordings. Thus, an open question remains as to how neural populations within the early vestibular pathways represent natural self-motion stimuli.

Accordingly, here we used multi-site probes to record the simultaneous activities of individual well-isolated vestibular-only neurons within the vestibular nuclei (henceforth referred to as vestibular nuclei neurons) during both naturalistic and artificial sinusoidal self-motion (see Materials and methods). We first investigated response heterogeneity. While vestibular nuclei neurons displayed similar dynamic tuning to self-motion, inspection of their spike trains revealed significant heterogeneity. Under sinusoidal stimulation, heterogeneity resulted equally from both trial-to-trial variability (i.e., different spike trains are elicited from a given neuron when presenting the same stimulus multiple times) and spiking variability across the vestibular nuclei neural population (i.e., different spike trains are elicited from different vestibular nuclei neurons during stimulation). In contrast, during naturalistic stimulation, heterogeneity primarily resulted from spiking variability. We next computed both signal and noise correlations (i.e., correlations due to the common stimulus versus those between the trial-to-trial variabilities of neural responses, respectively). Overall, while signal correlations were similar during both naturalistic and artificial stimulation, noise correlations were strikingly different. Specifically, the mean noise correlation was significantly positive during naturalistic but not artificial stimulation. Then, using computational modeling incorporating both heterogeneity and correlation seen experimentally, we show that heterogeneity and noise correlations during naturalistic stimulation are both beneficial for information transmission. Taken together, our results provide the first evidence that neurons within early vestibular pathways are adapted to the statistics of natural self-motion stimuli at the population level.

## Results

**Fig 1:**
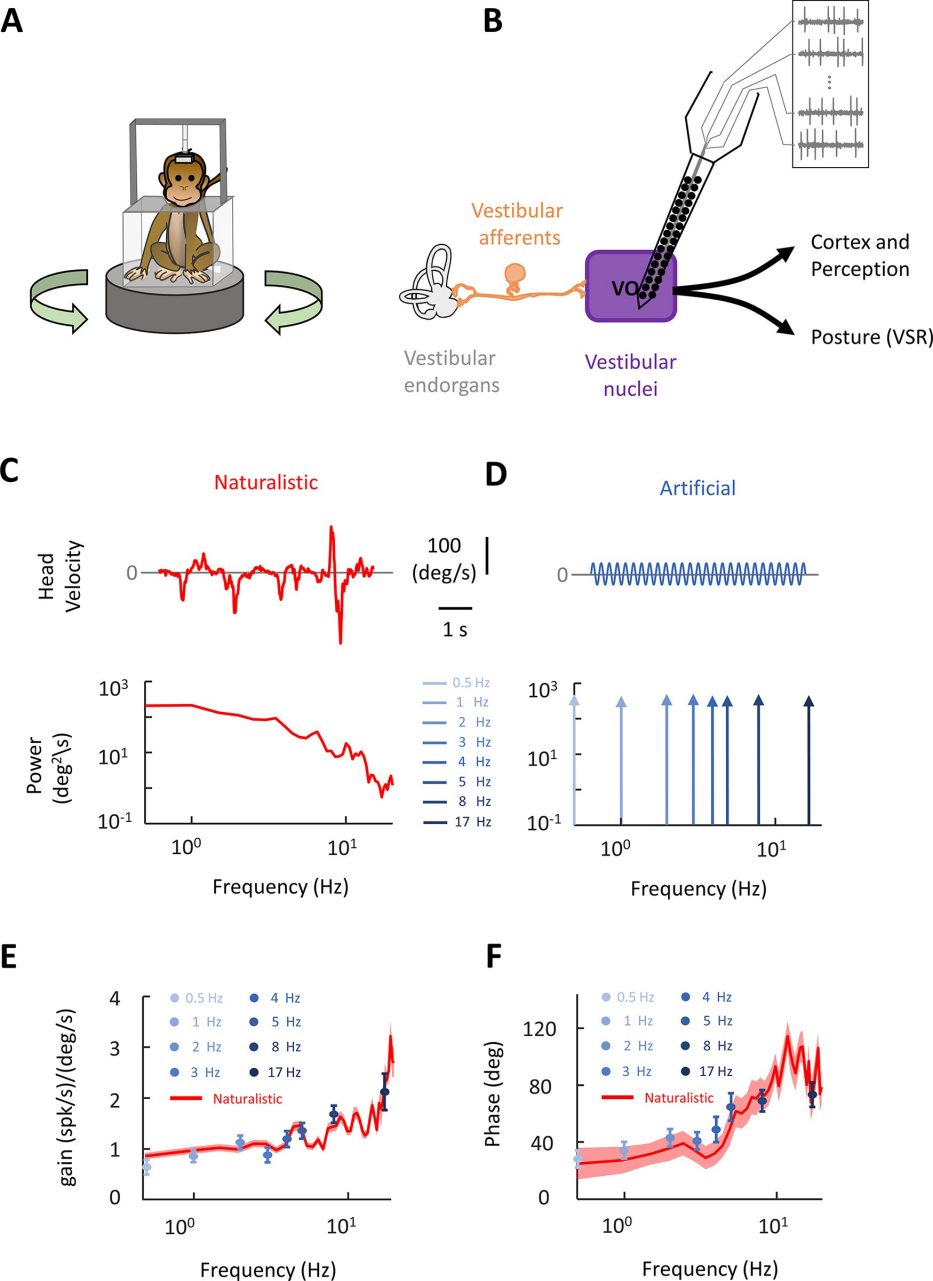
Experimental setup and stimuli used in this study. **(A)** A schematic of the experimental setup. During the experiments, the animal is head-fixed and seated comfortably on a turntable. **(B)** A schematic of early stages of the neural circuits involved in self-motion perception. Vestibular afferents transmit head motion information to VO neurons in vestibular nuclei which in turn project to spinal cord as well as thalamus and cortical areas and mediate postural reflexes and self-motion perception, respectively. A vector array probe was used to record neural activity of multiple VOs simultaneously in vestibular nuclei. **(C)** Head velocity traces and power spectra of the naturalistic stimulus used in the study. **(D)** Head velocity traces and power spectra of artificial stimuli used in the study. **(E)** Population-averaged gain for VO neurons during artificial stimuli (shown in shades of blue, similar to panel C; *N* = 42, f = 0.5 HZ; *N* = 42, f = 1 HZ; *N* = 40, f = 2 HZ; *N* = 41, f = 3 HZ; *N* = 40, f = 4 HZ; *N* = 41, f = 5 HZ; *N* = 39, f = 8 HZ; *N* = 37, f = 17 HZ) and naturalistic stimulus (shown in red; *N* = 41). Error bars and the error band show 1 SEM for artificial and naturalistic stimuli, respectively. **(F)** Population-averaged phase for VO neurons during artificial (shown in shades of blue, similar to panel C; *N* = 42, f = 0.5 HZ; *N* = 42, f = 1 HZ; *N* = 40, f = 2 HZ; *N* = 41, f = 3 HZ; *N* = 40, f = 4 HZ; *N* = 41, f = 5 HZ; *N* = 39, f = 8 HZ; *N* = 37, f = 17 HZ) and naturalistic stimulation (shown in red; *N* = 41). Error bars and the error band each show 1 SEM.

The goal of this study was to investigate how neural populations within the vestibular nuclei encode naturalistic self-motion. To address this question, we delivered stimuli to head-fixed *Macaca mulatta* that were comfortably seated on a turntable (Fig 1A) while recording the activities of vestibular-only neurons within the vestibular nuclei (Fig 1B). Our dataset comprised neural activities from 3 awake behaving animals for which we were able to record simultaneously from individual well-isolated single neurons during the highly dynamic self-motion stimuli described below (49 single neurons in total: 11 from monkey D; 34 neurons from Monkey B; 4 neurons from Monkey O; 35 pairs in total: 6 from monkey D; 26 from Monkey B; 3 from Monkey O).

Neurons were classified as either type 1 (*N* = 33) or 2 (*n* = 16) depending on whether they responded with increased firing rate to rotations towards the ipsilateral or contralateral sides, respectively (see Materials and methods). We note that these correspond to ON and OFF-type cells in other systems.

Self-motion stimuli consisted of rotations whose time course closely mimicked that recorded while the animal performed natural behaviors (e.g., walking, jumping) [48]. These stimuli are henceforth referred to as naturalistic (see Materials and methods and Fig 1C). For comparison, artificial self-motion stimuli consisting of sinusoids with amplitude 15 deg/s at frequencies 0.5, 1, 2, 3, 4, 5, 8, and 17 Hz were also used (Fig 1D). While naturalistic self-motion stimuli contain a spectrum of frequencies and can reach large amplitudes (approximately 200 deg/s) and, as such, strongly differ from sinusoids that each only contain 1 frequency and reach lower amplitudes (compare Fig 1C and 1D). Overall, the response profiles of single vestibular nuclei neurons in our dataset to sinusoidal were similar to those previously reported during both artificial and naturalistic stimulation as quantified by neural gain (Figs 1E, S1A and S1B) and phase (Figs 1F, S1C and S1D) [43–45,51]. These properties were consistent across all 3 animals (S2 Fig). Moreover, models based on gain and phase in general correctly predicted neural responses during naturalistic and artificial stimulation (S3 Fig).

### Vestibular nuclei neurons display heterogeneous spiking activities during naturalistic and artificial stimulation

**Fig 2:**
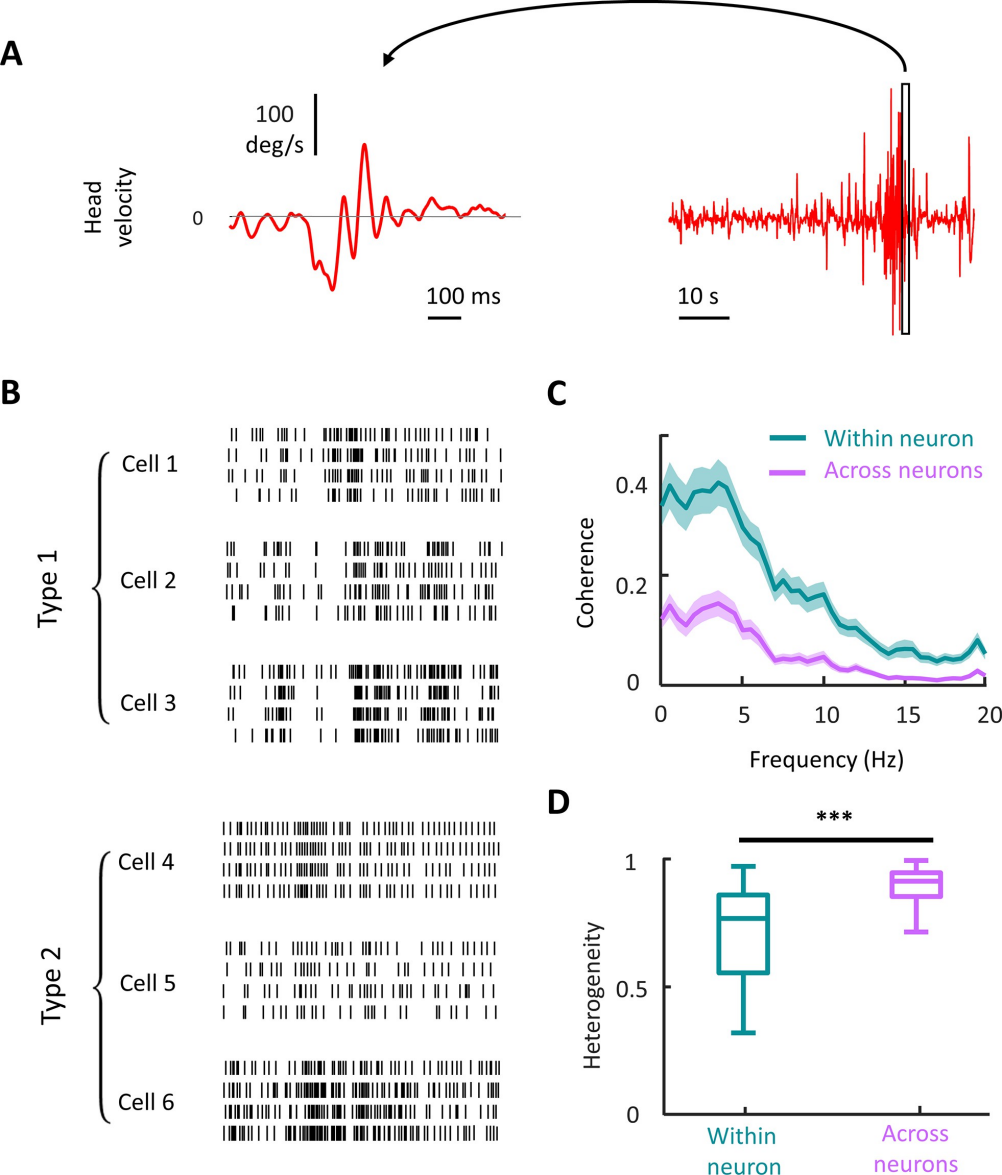
Vestibular nuclei neural responses are highly heterogeneous across neurons in the population during naturalistic stimulus. **(A)** Right: The entire waveform of naturalistic head velocity stimulus used in the study. Left: A 1 s snippet of the stimulus magnified to demonstrate the detailed temporal dynamics of the stimulus. **(B)** Raster plots for 3 exemplar type I and 3 exemplar type 2 VO neurons during the same stimulus snippet in panel A. The temporal scale bar is shared between panels A and B. **(C)** The population-averaged response–response coherence across neurons (magenta) and trials (turquoise) (*N* = 41). The error band shows 1 SEM. **(D)** The population-averaged heterogeneity in VOs is significantly higher across neurons than across trials (Wilcoxon rank sum test, *N* = 41, *p* = 5.6 × 10^−12^). We note that there are significantly more combinations across neurons than within neurons. The data for this panel are available from the Borealis database (https://doi.org/10.5683/SP3/FXFZ2J) (see files “Fig 2D.mat,” “Fig 2D.m,” and associated “readme.txt”).

We first investigated how neural populations within the vestibular nuclei responded to naturalistic versus artificial stimulation. During naturalistic stimulation (Fig 2A), we found that spiking responses were heterogeneous. This response heterogeneity resulted not only from different neurons displaying different spiking patterns (i.e., across neuron variability), but also a given neuron displaying different spiking patterns to repeated stimulus presentations (i.e., “trials”): this is henceforth referred to as trial-to-trial or within neuron variability (Fig 2B). Accordingly, we quantified the respective contributions of across neuron and within neuron variability towards determining heterogeneity by computing the response–response coherence [53] (see Materials and methods). The response–response coherence measures the similarity between neural responses and ranges between 0 (both responses are uncorrelated) and 1 (both responses are perfectly correlated up to a given time delay) [53]. We note that the coherence is insensitive to phase differences between spike trains and can be thought of a frequency-dependent correlation coefficient magnitude. Furthermore, we note that there are many more ways to combine activities across neuron than within neuron.

Overall, we found higher response–response coherence values when considering spiking responses from a given neuron to repeated stimulus presentations (i.e., within neuron; Fig 2C, turquoise) than when considering spiking responses from different neurons (i.e., across neurons; Fig 2C, magenta). We next quantified the contributions of across and within neuron variability to response heterogeneity (see Materials and methods). Overall, we found significantly higher values across neurons Wilcoxon rank sum test (*N* = 41 neurons, *p* = 5.6 × 10^−12^; Fig 2D), indicating that heterogeneity primarily results from across neuron variability.

**Fig 3:**
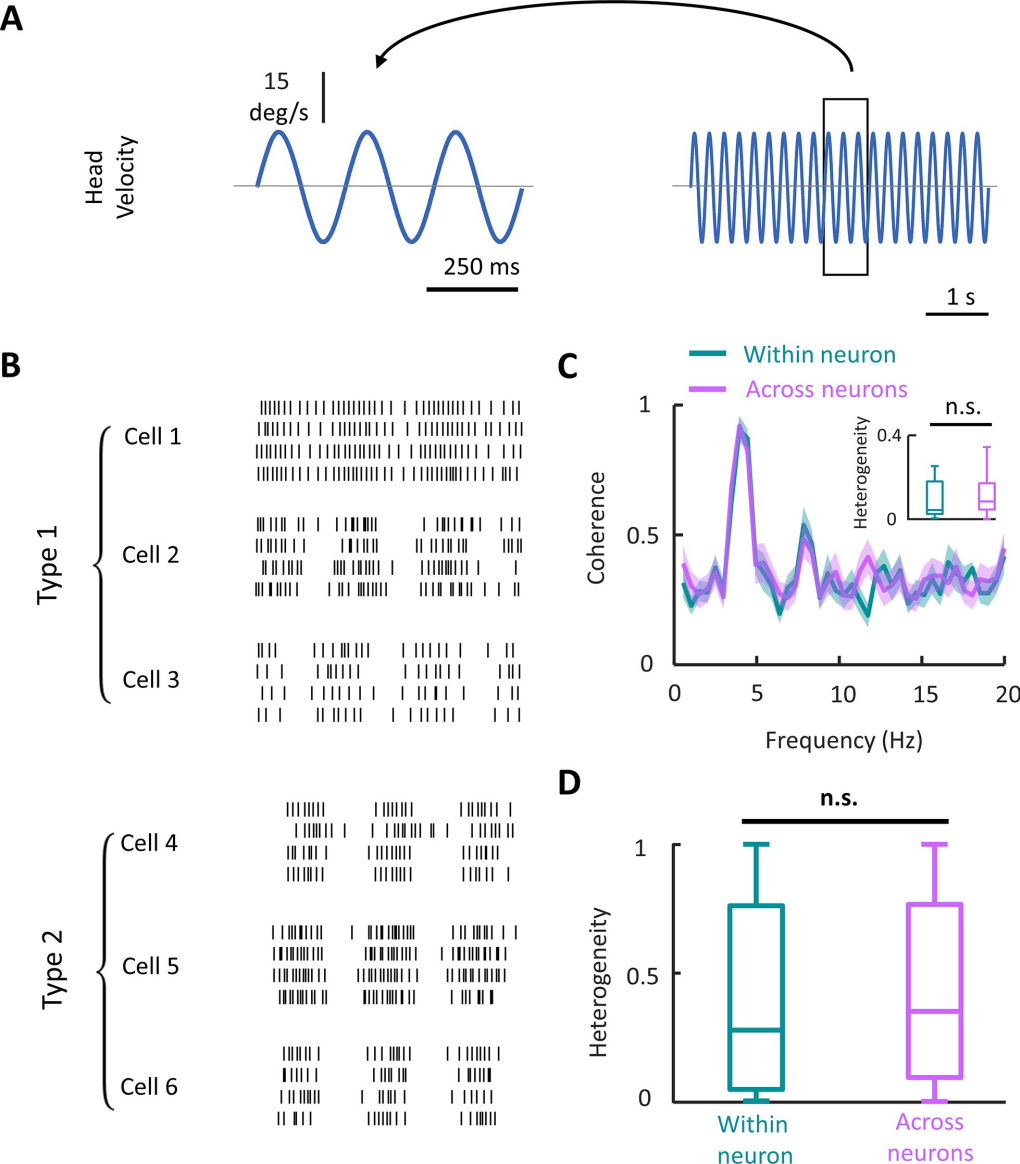
Vestibular nuclei neural responses are heterogenous during artificial stimulation. **(A)** Right: Example artificial sinusoidal head velocity stimulus (f = 4 Hz) used in the study. Left: A 3-cycle snippet of the stimulus. **(B)** Raster plot of 3 exemplar type I and 3 exemplar type 2 VO neurons during the same stimulus snippet in panel A. The temporal scale bar is shared between panels A and B. **(C)** The population-averaged response–response coherence across neurons (magenta) and trials (turquoise) during 4 Hz artificial stimulation. Inset: Boxplots showing within and across neuron variability during 4 Hz artificial stimulation (Wilcoxon rank sum test, *N* = 40, *p* = 0.099). The error band indicates 1 SEM. **(D)** The population-averaged heterogeneity in VOs is not significantly different across neurons and across trials (Wilcoxon rank sum test, *N* = 322, *p* = 0.26). The data for this panel are available from the Borealis database (https://doi.org/10.5683/SP3/FXFZ2J) (see files “Fig 3D.mat,” “Fig 3D.m,” and associated “readme.txt”).

We next investigated heterogeneity under artificial stimulation (Fig 3A) and obtained markedly different results. Specifically, neural spiking activities were heterogeneous but to a lesser extent than what was observed under naturalistic stimulation (compare Figs 2B and 3B). Quantification of across and within neuron variability as quantified by coherence revealed higher values than during naturalistic stimulation (compare Figs 2C, 3C, and S4). It is important to note that the coherence curves shown in Fig 3C were obtained during 4 Hz sinusoidal stimulation. As such, the peak at 4 Hz is expected as neurons modulated their firing rate in response to the stimulus (Fig 3B). Coherence curves obtained during sinusoidal stimulation at other frequencies are shown in S4 Fig. Interestingly, both within and across neuron variability made contributions to heterogeneity that were not significantly different from one another (Wilcoxon rank sum test, *N* = 40 neurons, *p* = 0.099; Fig 3C). Qualitatively similar results (i.e., no significant difference between heterogeneity computed either across or within neuron) were observed for all frequencies (S4 Fig) as well as when pooling across stimulation frequencies (Wilcoxon rank sum test, *N* = 322, *p* = 0.26, Fig 3D). This result shows that differences in the source of heterogeneity observed during naturalistic and artificial self-motion were robust to stimulation frequency.

### Correlations between vestibular nuclei neural activities differ during naturalistic versus artificial stimulation

We next investigated correlations between vestibular nuclei neural activities. As mentioned above, correlations can be separated between those that are due to the common stimulus (i.e., signal correlations) and correlations between the trial-to-trial variabilities of neural responses to repeated stimulus presentations (i.e., noise correlations). It is important to consider both signal and noise correlations since the relationship between these 2 measures (i.e., the correlation structure) is important for determining the effects of correlations on information transmission [8,9].

**Fig 4:**
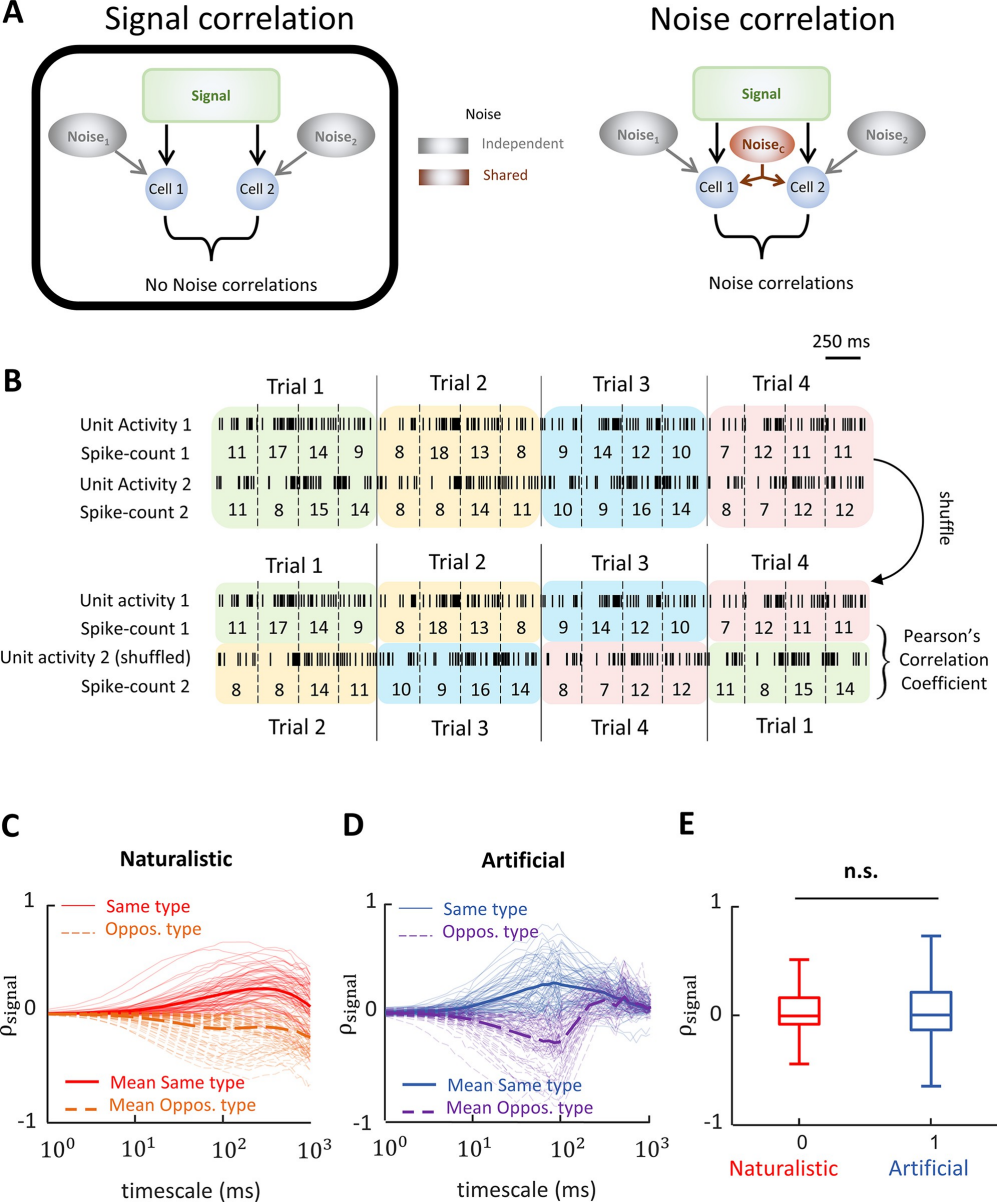
Signal correlations are similar during artificial and naturalistic stimulation. **(A)** Left: Schematic showing a hypothetical scenario in which the activities of 2 neurons are modulated by a common signal, which gives rise to signal correlations as well as by independent noise sources. In this case, because the noises are independent, there are no noise correlations. Right: Schematic showing a hypothetical scenario similar to the one described for panel A except that a source of shared noise has been added, which will give rise to noise correlations. Such shared noise could in principle originate from lateral connections as well as shared afferent input. **(B)** Methodology used to calculate signal correlation between the response of pair of VO neurons. The unit activity of cell 1 and cell 2 from Fig 2B is shown. Top: For each cell, the number of spikes is counted for a given timescale (e.g., 250 ms) and spike count sequences are generated. Bottom: The spike count sequence for the second neuron is shuffled to exclude the effect noise correlations due to simultaneous common input. The signal correlation for the timescale is calculated by computing Pearson’s correlation coefficient between the latter spike count sequences. **(C)** Signal correlations as a function of timescale during naturalistic stimulus (*N* = 820 pairs). The solid and dashed lines represent the correlations for the same-type and opposite-type pairs, respectively. The thick solid and dashed lines are the average values of the correlations for the same-type and opposite-type pairs, respectively. **(D)** Same as in C except it was calculated for an artificial stimulus (f = 4 Hz; *N* = 780 pairs). **(E)** Boxplots showing the signal correlation values during artificial (all frequencies) and naturalistic stimuli. Signal correlations during artificial and naturalistic stimuli are not significantly different (two-sample *t* test, *N* = 861 pairs, *p* = 0.83). For artificial stimuli, the timescale was chosen to be a quarter to a half-period of the sinewave period, where the signal correlations were maximum in magnitude on average. For naturalistic stimulus, the signal correlations were calculated for a 100 ms timescale that was consistent with the time scale of the stimulus and where signal correlations were highest in magnitude. In panels C and D, while the average values were calculated using all the pairs, only 75 traces of same and opposite-type pairs were shown for visualization purposes. Note that data were computed over different pairings during naturalistic and artificial stimulation, this is because not all pairs were held during both protocols. The data for this panel are available from the Borealis database (https://doi.org/10.5683/SP3/FXFZ2J) (see files “Fig 4E.mat,” “Fig 4E.m,” and associated “readme.txt”).

To quantify signal correlations (Fig 4A), we computed spike count sequences of pairs of vestibular nuclei neurons over trials (i.e., repeated presentations of the same stimulus waveform) using a time window with given length (i.e., “timescale”). We then shuffled the response across trials and then calculated Pearson’s correlation coefficients (Fig 4B; see Materials and methods for more details) [54], while systematically varying the timescale (1 to 1,000 ms). Fig 4C and 4D show signal correlations obtained during naturalistic and 4 Hz artificial stimulation, respectively. In both cases, signal correlations tended to be positive for same type (i.e., type 1-type 1 and type 2-type 2) pairs and negative for opposite (i.e., type 1-type 2) pairs. This is expected since type 1 and type 2 vestibular nuclei neurons respond with excitation to rotations towards the ipsilateral and contralateral sides, respectively. In the case of naturalistic stimulation, signal correlation magnitude was maximal for a timescale of approximately 250 ms, which corresponds to the correlation time of the stimulus (i.e., the time constant characterizing the stimulus’ autocorrelation exponential decay) [45]. This is because the stimulus varies maximally during that time. In the case of 4 Hz artificial stimulation, signal correlation magnitude was maximal for a timescale of approximately 100 ms, which corresponds roughly to the stimulus half-period. These results were expected as signal correlation magnitude will be maximal on timescales for which the stimulus varies the most. Qualitatively similar results were obtained for other frequencies (S5 Fig). Overall, signal correlations were similar during both naturalistic and artificial stimulation (Fig 4E; two-sample *t* test, *N* = 861 pairs, *p* = 0.83).

**Fig 5:**
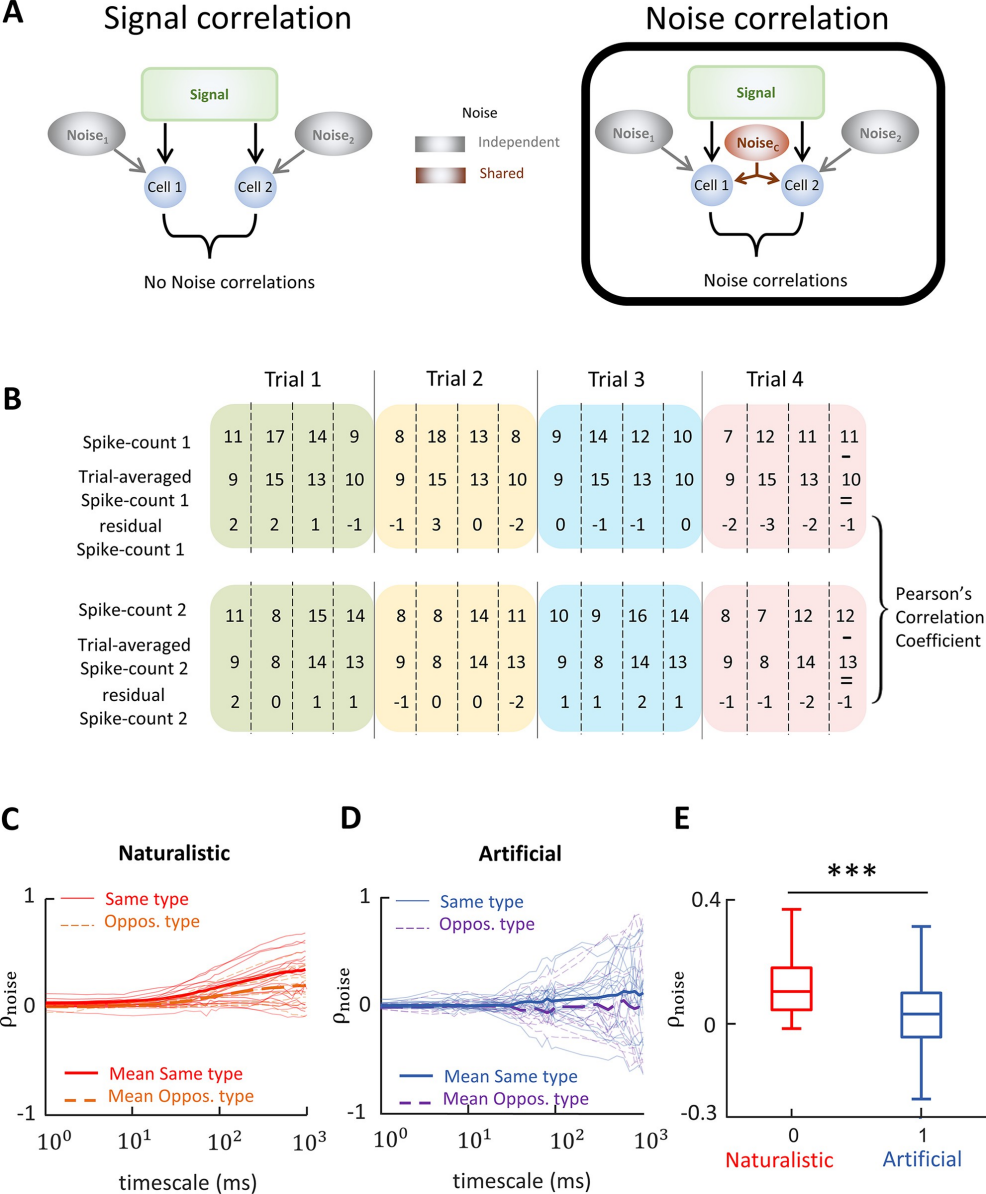
Noise correlations are significantly higher during naturalistic stimulus than during artificial stimulation. **(A)** Left: Schematic showing a hypothetical scenario in which the activities of 2 neurons are modulated by a common signal, which gives rise to signal correlations as well as by independent noise sources. In this case, because the noises are independent, there are no noise correlations. Right: Schematic showing a hypothetical scenario similar to the one described for panel A except that a source of shared noise has been added, which will give rise to noise correlations. Such shared noise could in principle originate from lateral connections as well as shared afferent input. **(B)** Methodology used to calculate noise correlation between the response of pair of VO neurons. The spike count sequences for the same cells (cell 1 and cell 2 in Fig 2B and 2D) as well as trial averaged spike counts using the same temporal window (timescale) are shown. For each cell, the residual spike count is calculated by subtracting the trial-averaged spike counts from raw spike counts. The noise correlation coefficient is calculated by computing the Pearson’s correlation coefficient of the residual spike count sequences. **(C)** Noise correlations as a function of timescale during naturalistic stimulus (*N* = 35 pairs). The solid and dashed lines represent the correlations for the same-type and opposite-type pairs, respectively. The thick solid and dashed lines are the average values of the correlations for the same-type and opposite-type pairs, respectively. **(D)** Same as in C except during 4 Hz artificial stimulation (*N* = 35 pairs). **(E)** Boxplots showing noise correlation values during artificial (all frequencies) and naturalistic stimuli. Noise correlations during naturalistic stimuli are significantly higher than that of during artificial stimuli (two-sample *t* test, *N* = 35 pairs, *p* = 3.2 × 10^−5^). The timescales at which the noise correlations were computed were the same as those used above for signal correlations. Note that data were computed over different pairings during naturalistic and artificial stimulation, this is because not all pairs were held during both protocols. The data for this panel are available from the Borealis database (https://doi.org/10.5683/SP3/FXFZ2J) (see files “Fig 5E.mat,” “Fig 5E.m,” and associated “readme.txt”).

We next quantified noise correlations (Fig 5A) by computing the Pearson’s correlation coefficient between the residual spike counts (i.e., the sequences obtained by subtracting the mean spike count across stimulus trials (Fig 5B; see Materials and methods for more details). Fig 5C and 5D show noise correlations obtained during naturalistic and 4 Hz artificial stimulation, respectively. Qualitatively different results were obtained in that, while noise correlations tended to be positive during naturalistic stimulation and were thus on average positive (Fig 5C), they instead tended to be both positive and negative during 4 Hz artificial stimulation, such that their average was zero (Fig 5D). Qualitatively similar results were obtained for other frequencies (S6 Fig). Thus, overall noise correlations were significantly more positive during naturalistic than during artificial stimulation (two-sample *t* test, *N* = 35, *p* = 3.2 × 10^−5^; Fig 5E).

It is important to note that changes in noise correlations were most likely not due to differences in amplitude between naturalistic and artificial stimulation, as noise correlations were also positive on average when restricting our analysis to segments of naturalistic stimulation with low amplitude (S7 Fig). Moreover, changes in noise correlations were not due to differences in firing rate, as neurons displayed similar firing rates during artificial and naturalistic stimulation (S8 Fig). As such, we hypothesize that increased noise correlations are caused by the characteristic frequency spectrum displayed by naturalistic but not artificial stimuli. This is further discussed below. Finally, noise correlations were significantly more positive during naturalistic stimulation relative to artificial stimulation for a wide range of timescale (all timescales between 7 ms and 1 s; *p* < 0.03 in all cases).

### Increased noise correlations during naturalistic stimulation benefits population coding by heterogeneous vestibular neural populations

Our results so far show that the spiking activities of vestibular nuclei neurons are more heterogeneous during naturalistic than during artificial stimulation. Such changes were accompanied by changes in noise correlations, which were more positive during naturalistic stimulation. Such a change in noise correlations might seem surprising at first glance as previous studies have shown that this can increase redundancy and thus impair information transmission [55]. It should be however noted that these assumed a homogeneous population, whereas our results show that this is not the case for vestibular nuclei neurons.

**Fig 6:**
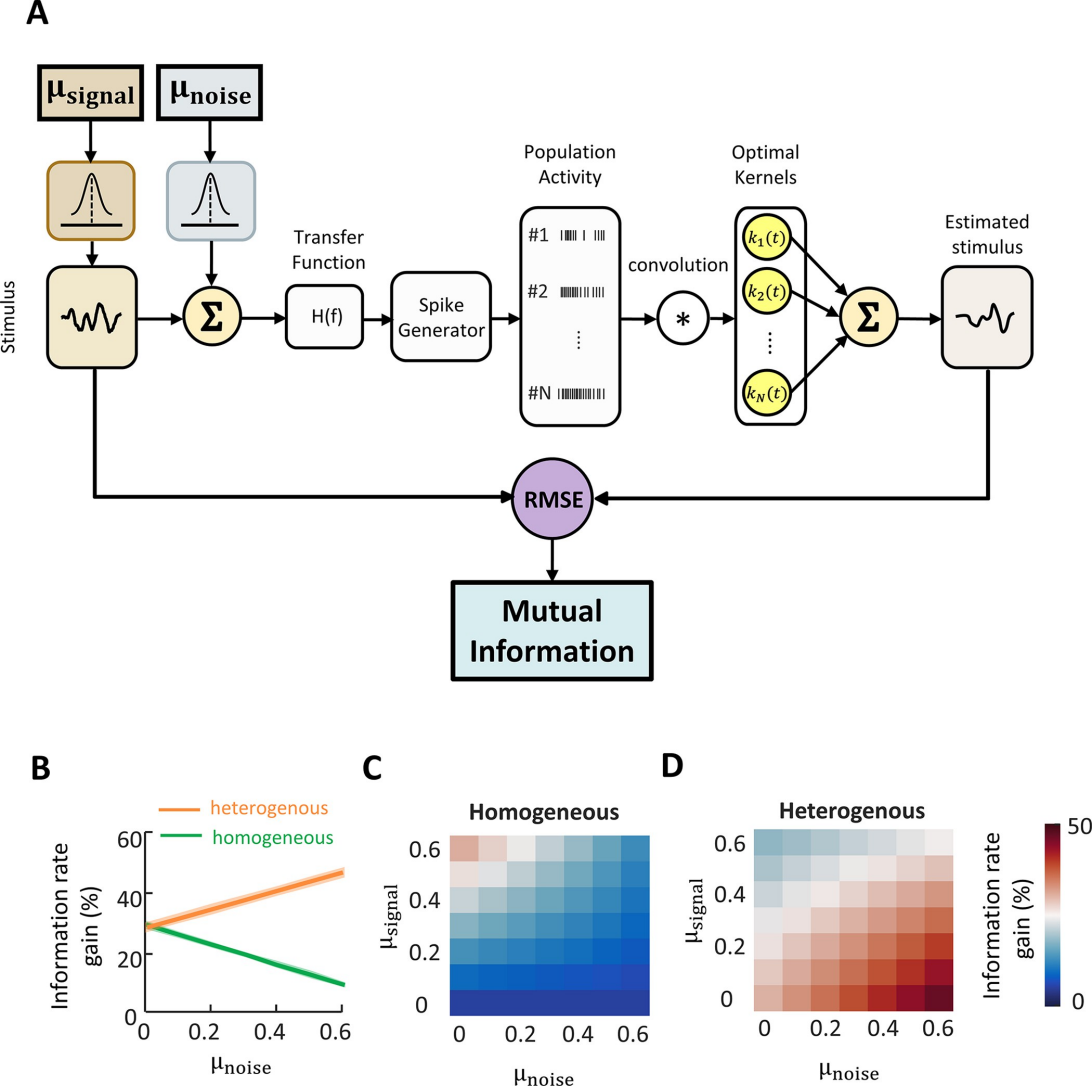
Noise correlations benefit population coding in heterogenous neural populations but reduce information in homogenous neural populations. ** (A)** Schematic of a model decoding head velocity information from population of simulated VO neurons with a given correlation structure. For a given mean noise (ρ_noise_) and signal (ρ_signal_) correlation, a narrow (homogenous; σ = 0.03) or wide (heterogenous; σ = 0.15) distribution of correlation coefficients as well as the corresponding correlation matrices are generated. The correlation matrices are used to generate correlated signal and noise inputs to spike generator component (i.e., the LIF model), the output of which are correlated with the desired correlation structure. The generated spikes are used to estimate a set of kernel functions that minimize MSE between the stimulus and its estimation. The mutual information is then calculated from the decoded signal. **(B)** Information gain rate as function of mean noise correlation for heterogenous (ρ_signal_ = 0) and homogenous conditions for mean signal correlation ρ_signal_ = 0.6. Error bands indicate 1 SEM (*N* = 40 simulations). Note that the error bands are quite small. **(C)** Mutual information rate gain as a function of mean signal and noise correlation for homogenous population coding. The information rate gain is calculated by comparing the information in a given condition to the minimum information decoded across all simulated conditions (i.e., ρ_noise_ = 0.6, ρ_signal_ = 0 under homogenous conditions) (*N* = 40 simulations). **(D)** Same as in C, except the simulations are done for a heterogenous population activity (*N* = 40 simulations). LIF, leaky integrate-and-fire; MSE, mean squared error; SEM, Standard error of the mean; VO, vestibular-only.

Accordingly, in order to gain understanding as to how heterogeneity and correlations influence population coding, we built a computational model of vestibular nuclei neural population that incorporated their known tuning properties (Fig 6A; see Materials and methods). We then considered both homogeneous and heterogeneous populations and systematically varied both signal and noise correlations (see Materials and methods). Each neuron was modeled using a transfer function as well as a leaky integrate-and-fire (LIF) model for spike generation. We used the same naturalistic stimulus waveform as for our experimental data which was multiplied by a covariance matrix to generate correlated signal inputs (see Materials and methods). Furthermore, a set of white Gaussian noise was generated and correlated using a noise covariance matrix. We then quantified the mutual information from the model neural population’s performance at reconstructing the detailed time course of the stimulus. Our assumption that downstream brain areas decode information about the stimulus’ detailed time course from VN neural populations is justified as previous studies have shown that the activity of vestibular neurons in VPL receiving direct input from vestibular nuclei neurons faithfully follows the naturalistic stimulus’ detailed time course [51].

Overall, we found that, for given signal correlations, increasing noise correlations led to differential effects on information transmission for homogeneous (Fig 6B and 6C) and heterogeneous (Fig 6B and 6D) populations. For homogeneous populations, the highest information was obtained when signal correlations were maximal and noise correlations near zero (Fig 6C). Increasing noise correlations led to a decrease in mutual information irrespective of signal correlations by increasing redundancy. This result is consistent with those of previous studies [55]. In contrast, for heterogeneous populations, the highest information was obtained when signal correlations are near zero and noise correlations highest (Fig 6D). As such, for given signal correlations, increasing noise correlations led to increased information for heterogeneous populations but instead led to decreased information for homogeneous populations (Fig 6B).

It is important to note that we took into account the fact that the slope of the linear relationship between noise and signal correlations (i.e., the “correlation slope”) can strongly influence information transmission [9,56,57]. Specifically, our experimental data shows that there was a significantly positive correlation slope during naturalistic but not artificial stimulation (S9 Fig). Thus, the correlation slope for heterogeneous populations was similar to that obtained during naturalistic stimulation while, for homogeneous populations, the correlation slope was instead similar to that obtained during artificial stimulation. Overall, the positive correlation slope reduced information transmission but did not affect the qualitative nature of our results (i.e., that information transmission was overall higher for heterogeneous populations; S10 Fig). Moreover, both coding fraction and correlation coefficients exhibited higher values for heterogenous populations (S11 Fig).

**Fig 7:**
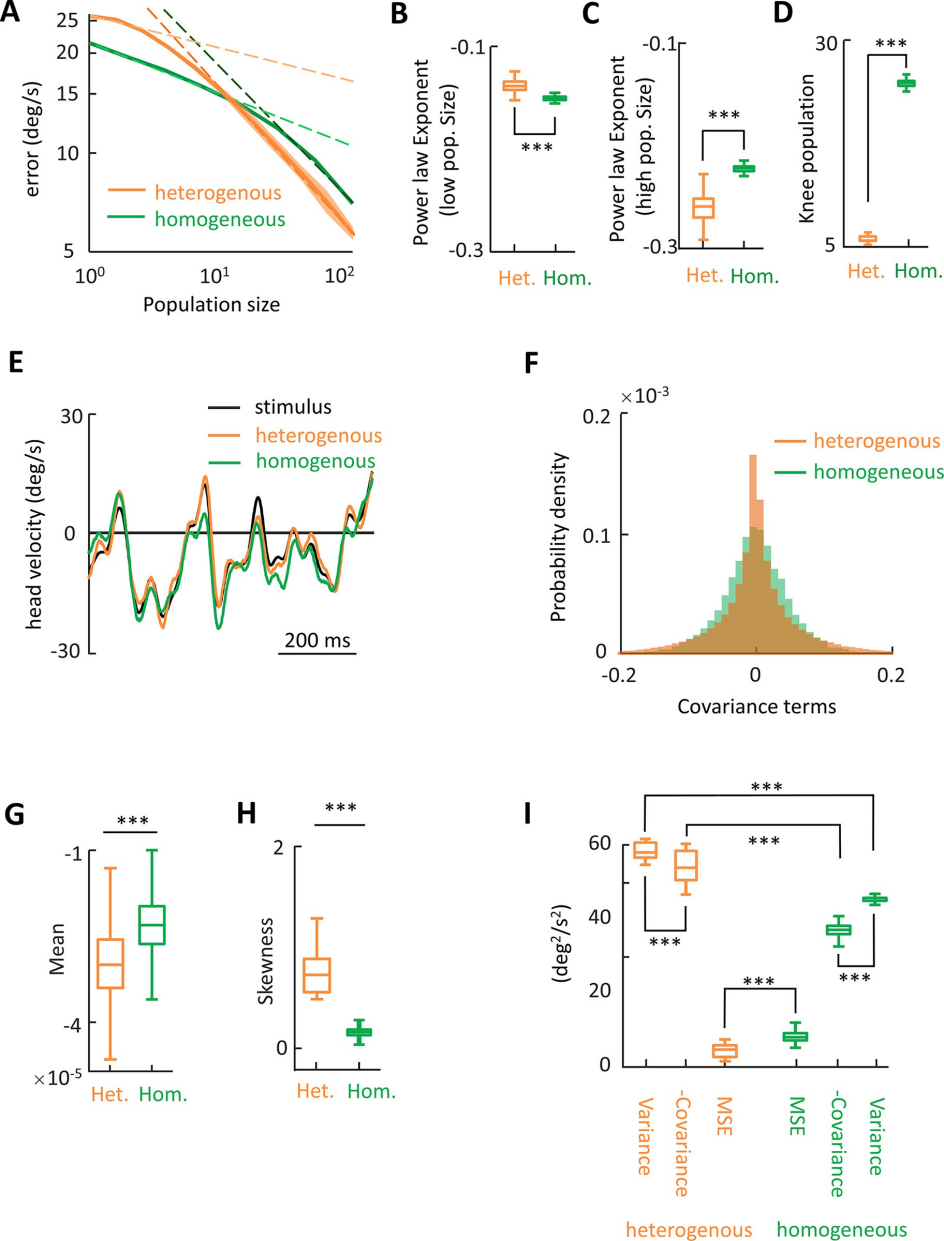
Heterogenous populations predict stimulus time course more accurately than homogenous populations. **(A)** Estimation error as a function of population size for populations with optimum correlation structure for heterogenous (ρ_noise_ = 0.6, ρ_signal_ = 0) and homogenous (ρ_noise_ = 0, ρ_signal_ = 0.6) conditions. Error bands indicate 1 SEM. Each curve is fitted with a double power law functions over the low and high population sizes, indicated with dashed lines. **(B)** The magnitude of power law exponents below the knee population sizes is significantly higher for heterogenous conditions (Wilcoxon rank sum test, *N* = 1,000 simulations, *p* = 2.2 × 10^−29^). The data for this panel are available from the Borealis database (https://doi.org/10.5683/SP3/FXFZ2J) (see files “Fig 7B.mat,” “Fig 7B.m,” and associated “readme.txt”). **(C)** The magnitude of power law exponents above the knee population sizes is significantly lower for heterogenous conditions (Wilcoxon rank sum test, *N* = 1,000 simulations, *p* = 3.9 × 10^−34^). The data for this panel are available from the Borealis database (https://doi.org/10.5683/SP3/FXFZ2J) (see files “Fig 7C.mat,” “Fig 7C.m,” and associated “readme.txt”). **(D)** The knee populations size for heterogenous conditions is significantly lower than that of homogenous condition (Wilcoxon rank sum test, *N* = 1,000 simulations, *p* = 2.6 × 10^−34^). The data for this panel are available from the Borealis database (https://doi.org/10.5683/SP3/FXFZ2J) (see files “Fig 7D.mat,” “Fig 7D.m,” and associated “readme.txt”). **(E)** Stimulus and reconstructed stimulus from the same heterogenous and homogenous conditions as in 7A (*N* = 128). **(F)** The distribution of covariance terms of the covariance matrices computed from reconstruction error signals during the same heterogenous and homogenous conditions as in 7A. **(G)** Mean of the covariance terms for heterogenous condition is significantly lower than that of homogenous condition (Wilcoxon rank sum test, *N* = 1,000 simulations, *p* = 1.9 × 10^−6^). The data for this panel are available from the Borealis database (https://doi.org/10.5683/SP3/FXFZ2J) (see files “Fig 7G.mat,” “Fig 7G.m,” and associated “readme.txt”). **(H)** The skewness of the distribution for the heterogenous and homogenous conditions (Wilcoxon rank sum test, *N* = 1,000 simulations, *p* = 1.8 × 10^−158^). The heterogenous population is skewed towards negative values and therefore has negative mean value. The data for this panel are available from the Borealis database (https://doi.org/10.5683/SP3/FXFZ2J) (see files “Fig 7H.mat,” “Fig 7H.m,” and associated “readme.txt”). **(I)** Summed variance terms (Var.), summed covariance terms (Cov.), and MSE for covariance matrices computed from reconstruction error signals for heterogenous and homogenous conditions. All quantities compared are significantly different than each other (Wilcoxon rank sum test, *N* = 1,000 simulations, *p* = 5.3 × 10^−7^). The data for this panel are available from the Borealis database (https://doi.org/10.5683/SP3/FXFZ2J) (see files “Fig 7I.mat,” “Fig 7I.m,” and associated “readme.txt”). MSE, mean squared error.

Why do increased noise correlations benefit information transmission for heterogeneous but not homogeneous neural populations? To answer this important question, we plotted the reconstruction error as a function of population size (Fig 7A). Overall, in both cases, reconstruction error decreased initially more slowly and then faster with increasing population (Fig 7A). As such, the curves were fitted using 2 different power laws for low and high population sizes (see Materials and methods). Overall, the power law exponent for high population sizes was significantly more negative for a heterogeneous than for a homogeneous population (*p* = 3.9 × 10^−34^), whereas the opposite was observed for power law coefficients concerning low population sizes (*p* = 2.2 × 10^−29^; Fig 7B and 7C). Moreover, the “critical population size” at which this transition occurred was lower for heterogeneous populations (*p* = 2.6 × 10^−24^; Fig 7D). Thus, for large enough population size (e.g., *N* = 128), the reconstructed stimulus is more similar to the original stimulus for the heterogeneous population (Fig 7E).

To understand why the reconstruction error is smaller in the heterogeneous case for sufficiently large population sizes (e.g., compare orange and green curves in Fig 7A for a population size of 100), we considered the respective contributions of individual model neurons (see Materials and methods). Specifically, the reconstruction error can be written as a sum of “variance” (i.e., within neuron) and “covariance” (i.e., across neurons) terms. While variance terms are positive by definition, covariance terms can be negative and thus contribute to reducing the overall error. Our results show that the distribution of covariance terms was more skewed towards negative values for heterogeneous populations and was thus negative on average (Fig 7F, 7G, and 7H). In contrast, the distribution of covariance terms for the homogeneous population was more symmetric and displayed an average that was closer to zero (Fig 7F, 7G, and 7H). Quantification of mean variance terms revealed overall higher variances for heterogeneous as compared to homogeneous populations (*p* = 2.1 × 10^−8^, Kruskal–Wallis test; Fig 7I). Thus, the covariance terms better subtract from the variance terms for the heterogeneous case, thereby leading to a lower reconstruction error (*p* = 2.1 × 10^−8^, Kruskal–Wallis test; Fig 7I).

An important question is then, why is the distribution of covariance terms more skewed for heterogeneous than for homogeneous neural populations? Overall, this implies that the reconstruction errors of individual neurons tend to be more negatively related to each other in heterogeneous populations. This is because signal correlations were on average zero and noise correlations positive to obtain maximal information for heterogeneous populations (Fig 6D). Thus, signal correlations were sometimes negative while noise correlations were sometimes positive, which greatly benefits information transmission by effectively reducing variability beyond that achievable when noise correlations are zero [9]. In contrast, for homogeneous populations, maximal information was obtained when signal correlations were positive and noise correlations near zero on average (Fig 6C). In this case, variability is not as effectively reduced, leading to reconstruction errors that are more positively related and higher than for the heterogeneous case.

## Discussion

### Summary of results

Here, we investigated how vestibular nuclei neural populations within the vestibular nuclei represent naturalistic as opposed to artificial self-motion stimuli. We found that, while vestibular nuclei neurons displayed similar dynamic tuning during both stimulation conditions, their spiking activities were heterogeneous (i.e., spike trains elicited by the same stimulus were different from one another). Under artificial stimulation, heterogeneity resulted from trial-to-trial variability in individual neurons as well as from variability across neurons. In contrast, under naturalistic stimulation, heterogeneity resulted primarily from variability across neurons and was higher than that observed during artificial stimulation. We then investigated correlations between vestibular nuclei neural activities. While signal correlations were similar during both stimulation conditions, noise correlations were significantly more positive during naturalistic stimulation. Further computational modeling revealed that heterogeneity and noise correlations during naturalistic stimulation were both beneficial for information transmission. Taken together, our results demonstrate for the first time that neurons in early vestibular pathways are adapted to the statistics of natural self-motion stimuli at the population level.

### Stimulus-dependent noise correlations in vestibular nuclei neural populations: Implications for coding

Our findings show that vestibular nuclei neural populations display qualitatively different correlation structures under naturalistic and artificial stimulation. Specifically, during naturalistic stimulation, noise correlations were significantly positive and had a beneficial effect on information transmission when taking into account response heterogeneity among vestibular nuclei neurons. In contrast, under artificial stimulation, noise correlations were on average close to zero and had minimal effect on information transmission. This latter result is consistent with the findings of the only prior study to have investigated population coding in ascending vestibular pathways which focused solely on artificial low frequency stimuli [46]. Thus, taken together, our results establish that noise correlations between vestibular nuclei neural activities are stimulus-dependent.

How noise correlations impact population coding of natural versus artificial stimuli has been investigated in other sensory systems, including the visual, auditory, and electrosensory systems. Unlike the coding properties of individual neurons which can strongly differ between conditions, these results of these prior studies have shown that distribution of noise correlations and their effects on population coding are generally similar. Notably, on average, noise correlations are close to zero and have, at best, minimal effects on information transmission during conditions designed to evoke more natural stimulation (visual: [58,59]; auditory: [60,61]; electrosensory: [62,63]) as well as in artificial stimulation conditions (see [1,8,9,64] for review).

At first glance, these findings may appear to be at odds with our present results that have demonstrated positive noise correlations, which are significant under naturalistic self-motion stimulation and benefit information transmission. However, it should be emphasized that, the conditions used in these prior studies did not evoke fully natural stimulation. For example, prior studies of the visual system used stimuli that were natural in the spatial domain, but not in the temporal domain. This is because sequences of natural images were played in a pseudorandom fashion with a given image only presented for a short time (100 to 200 ms) [58,59]. As a result, the temporal dynamics of the stimuli used did not resemble those experienced during natural everyday behaviors, which could contribute to the differences observed in these prior studies versus our present results. Moreover, in the case of the auditory and electrosensory systems, the natural stimuli used displayed frequency contents that were much higher (>10 kHz) than that of natural self-motion stimuli used in the current study (0 to 20 Hz) (see [49] for review), which may also contribute to the observed differences as the greater frequency bands would then have to be normalized to the biologically relevant range. Further studies are needed to test these predictions.

It should be further noted that one feature of natural auditory and electrosensory stimuli, the time-varying amplitude or envelope which carries important information [65,66], tends to contain much lower temporal frequencies (<100 Hz) [67,68] that better overlap with that of natural self-motion stimuli and thus could elicit noise correlations. We speculate that results similar to our own will be obtained when investigating population coding of natural sound envelopes within ascending auditory and electrosensory pathways. Further studies are needed to test these predictions as our current understanding of natural envelope coding in the auditory and electrosensory systems is based on single-unit recordings (see, e.g., [66,69]).

Finally, the statistics of natural stimuli change on multiple timescales, such that sensory systems must continuously adapt in order for coding to remain optimal [70–72]. Several studies have shown that population coding adapts to changes in natural stimulus statistics across sensory modalities (visual: [73]; auditory: [72]). However, how population coding of naturalistic self-motion stimuli adapts to changes in statistics has not been investigated to date. We predict that both heterogeneity and noise correlations should adapt to natural self-motion stimulus statistics to optimize coding. Future studies are needed to test these predictions.

### What is the origin of noise correlations among vestibular nuclei neurons during naturalistic versus artificial stimulation?

Our results show that noise correlations are significantly positive during naturalistic but not artificial self-motion stimulation. An open question is thus: Why are noise correlations in early vestibular pathways stimulus-dependent? Previous studies have shown that correlation magnitude is strongly dependent on response nonlinearities [29]. Accordingly, one possibility is that stimulus-dependent noise correlations result from naturalistic and artificial stimuli differentially eliciting response nonlinearities in vestibular nuclei neurons. However, naturalistic self-motion stimuli display higher amplitudes than artificial self-motion stimuli (see [49] for review). Therefore, in the present study, stronger response nonlinearities would have been elicited during naturalistic versus artificial stimulation, resulting in decreased rather than increased correlation magnitude. Thus, the potential explanation that the observed stimulus dependence of noise correlation magnitude is due to response nonlinearities cannot explain our experimental findings.

Another potential explanation for the experimentally observed stimulus dependence of noise correlations in early vestibular pathways is that vestibular nuclei neurons receive differential synaptic input during naturalistic and artificial stimulation. Specifically, it is conceivable that the relative balance between excitation and inhibition, which is critical towards determining correlation magnitude (see [34] for review), differs between both stimulation conditions. In this context, increased noise correlation magnitude during naturalistic stimulation could result from increased excitation due to better activation of gap junctions between neighboring vestibular nuclei neurons [74,75], which would increase synchrony. However, the fact that vestibular nuclei neurons display similar response dynamics to both artificial and naturalistic self-motion stimuli [45,51] (Fig 1D) suggests that this is not the case.

Alternatively, differential synaptic input could be due to activation of feedback from cortical areas onto vestibular nuclei neurons [76] during naturalistic stimulation. This later hypothesis is supported by the fact that recent results have shown that vestibular sensitive neurons within the posterior ventral lateral (VPL) area of the thalamus that project to cortex display different response dynamics to naturalistic and artificial self-motion stimuli [51]. Specifically, the activities of these neurons faithfully follow the time course of naturalistic but not artificial stimuli. Further studies are needed to understand the nature of the mechanisms mediating changes in noise correlations within the vestibular nuclei neural populations during naturalistic and artificial self-motion stimulation.

### Coding of self-motion by vestibular nuclei neural populations: Implications for self-motion perception

In addition to the observed differences in noise correlations, our present results show that vestibular nuclei neurons display heterogeneities in their spiking activities under naturalistic and artificial stimulation. Spiking heterogeneity has been observed ubiquitously across systems and species [77–81] and is thought to be beneficial for information transmission [15,18,22–24,82–84]. Computational modeling has revealed that higher information rates are consistently observed for heterogeneous populations irrespective of correlation structure, which occurs in addition to the beneficial effects of positive noise correlations mentioned above. Thus, increased noise correlations observed during naturalistic self-motion stimulation constitute an adaptation to natural stimulus statistics such as to increase information transmission by heterogeneous vestibular nuclei neural populations. As such, our results provide the first evidence that population coding within early vestibular pathways is adapted to natural stimulus statistics by increasing information transmission via the joint effects of heterogeneity and noise correlations.

We showed that changes in noise correlations are not due to differences in amplitude between naturalistic and artificial stimuli. Rather, they suggest that these changes are due to the characteristic frequency spectrum of natural self-motion stimuli. Further studies using stimuli where the frequency spectrum is systematically varied are needed to test this hypothesis. Importantly, the fact that noise correlations were significantly higher during naturalistic stimulation for a wide range of timescales shows robustness. Ultimately however, whether increased information due to heterogeneity and noise correlations is useful to the organism will depend on how information transmitted by VO neural populations are decoded by downstream brain areas. This is further discussed below. We moreover note that our model considered pairwise interactions between neural activities as well as tuning properties of VO neural populations. It is conceivable that other aspects of tuning (e.g., dynamic nonlinearities) as well as higher order interactions could potentially further influence information transmission [9]. Further studies are needed to test these predictions.

Vestibular-only neurons within the vestibular nuclei project directly to the VPL area of the thalamus (VPL) and are thus a key input to the posterior thalamocortical vestibular pathway [40]. As mentioned above, VPL neural activity faithfully follows the detailed time course of naturalistic but not artificial self-motion stimuli [51]. This finding implies that information transmitted about the stimulus’ detailed time course by vestibular nuclei neural populations is likely decoded by target neurons in the VPL. In turn, neurons in VPL project to multiple cortical areas such as the parietoinsular vestibular cortex (PIVC), the ventral intraparietal (VIP) cortex, area 2v of the intraparietal sulcus, and area 3a in the sulcus centralis [76,85–87]. In particular, integration of vestibular with extravestibular information in both PIVC and VIP plays a key role in our perception of self-motion and spatial orientation [88–90].

To date, vestibular neurons within both PIVC and VIP have only been tested using artificial stimuli [91–94]. Thus, how these neurons respond to natural self-motion is not known. We speculate that increased vestibular information transmitted by vestibular nuclei neural populations during naturalistic stimulation is decoded by such cortical areas to improve self-motion perception relative to artificial stimulation. Specifically, we predict that vestibular perceptual detection (i.e., the minimum stimulus amplitude that can be detected) and discrimination (i.e., the minimum change in stimulus amplitude that can be detected) thresholds should be significantly lower during naturalistic than during artificial self-motion stimulation. Further work is needed to test these predictions as all previous studies of vestibular perception have used artificial stimuli (see, e.g., [95–99]).

## Materials and methods

### Ethics statement

All procedures including surgeries, experiments, and housing of the animals were approved by the McGill University Animal Care Committee (protocol #4096) and in accordance with the guidelines of the Canadian Council on Animal Care. Experiments were conducted on 2 male (Monkey D aged 8 years, 7.1 kg; Monkey O aged 6 years, 6.3 kg) and 1 female (Monkey B, aged 10, 11.3 kg) rhesus macaques (*Macaca mulatta*). The animals were transferred to laboratories where extracellular recordings were conducted in a 2-h session for the experiments. After the experiment, the animals were returned to their housing units.

### Surgical procedure

Aseptic surgeries in preparation for extracellular recording and eye movement measurement were performed as described previously [44,51,100]. Briefly, animals were pre-anesthetized with ketamine hydrochloride (15 mg/kg im) and injected with buprenorphine (0.01 mg/kg im) and diazepam (1 mg/kg im) to provide analgesia and muscle relaxation, respectively. Loading doses of dexamethasone (1 mg/kg im) and cefazolin (50 mg/kg iv) were administered to minimize swelling and prevent infection, respectively. Anticholinergic glycopyrrolate (0.005 mg/kg im) was also preoperatively injected to stabilize heart rate and to reduce salivation, and then every 2.5 to 3 h during surgery. During surgery, anesthesia was maintained using isoflurane gas (0.8% to 1.5%), combined with a minimum 3 l/min (dose adjusted to effect) of 100% oxygen.

Heart rate, blood pressure, respiration, and body temperature were monitored throughout the procedure. During the surgical procedure, a titanium post for head immobilization and a titanium recording chambers that allowed access to the vestibular nucleus (VN) were fastened to each animal’s skull with titanium screws and dental acrylic over the abducens nucleus (−23.85 mm posterior from bregma and 2 mm lateral from midline) [101]. Craniotomy was performed within the recording chamber to allow electrode access to the brain stem. An 18-mm diameter eye coil (3 loops of Teflon-coated stainless-steel wire) was implanted in 1 eye behind the conjunctiva [102]. Following surgery, we continued dexamethasone (0.5 mg/kg im; for 4 days), anafen (2 mg/kg day 1, 1 mg/kg on subsequent days), and buprenorphine (0.01 mg/kg im; every 12 h for 2 to 5 days). In addition, cefazolin (25 mg/kg) was injected twice daily for 10 days. Animals recovered in 2 weeks before any experimenting began.

### Data acquisition

During the experiment, animals were head-fixed and seated comfortably on a primate chair mounted on a motion platform that delivered head motion stimuli during passive whole-body rotation (pWBR). For electrophysiological recordings, a neural probe (NeuroNexus vector array, Ann Arbor, Michigan, United States of America) with 32 recording sites was used to collect extracellular recording data from multiple individual neurons at a time (Shank thickness 75 μm; area of each recording site 177 μm^2^; vertical distance between sites 50 μm; horizontal distance between sites 43.3 μm; see S12A Fig). The probe was inserted in a guide tube and directed towards the vestibular nuclei. The angular velocity and gaze position signals were measured using a gyroscope and a magnetic search coil, respectively, and the signals were low pass filtered at 125 Hz and sampled at 1,000 Hz. Extracellular recording data were band-pass filtered at 300 to 3,000 Hz, sampled at 30 kHz, and collected via Cerebus Neural Signal Processor (Blackrock Systems).

### Experimental paradigm

Localization of vestibular nuclei was done relative to the abducens nucleus based on the discharge characteristic of the abducens neurons—the “singing beehive” sound—during spontaneous eye movements. To identify vestibular-only neurons within the vestibular nuclei, isolated cells were tested for vestibular sensitivity and lack of eye movement sensitivity by training the animals to perform smooth pursuit, saccades, and vestibulo-ocular reflex cancellation (VORc) prior to the recordings. Specifically, a fixed target was displayed at the center of a screen in front of the animal. After the fixation period, saccadic eye movements were elicited via the presentation of a target at positions ±10°, ±20°, and ±30°. Smooth pursuit movements were elicited by moving a target sinusoidally on the screen with a span of ±30°.

Each recorded neuron’s firing rate was then quantified for different eye positions as done previously and only neurons for which there was no significant correlation between firing rate and eye position were retained [44]. The monkeys performed VOR by fixating on a fixed target at the center of the screen during 0.5 Hz sinusoidal pWBR. Moreover, the animals performed VORc during 0.5 Hz pWBR while fixating on a target on the screen which moved synchronously with the primate chair and animal. Recordings were initiated after isolating at least 2 vestibular nuclei neurons based on lack of sensitivity to eye movements as described below.

### Stimulus protocol

Following the characterization of the neurons, an interval of 20 s was used to record the resting discharge activity of the neurons. Next, sinusoidal stimuli with maximum angular velocities of 15 deg/s and frequencies of 0.5, 1, 2, 3, 4, 5, 8, and 17 Hz were delivered to the animal. Each stimulus was presented at least for a minimum of 10 cycles (i.e., trials). Neural responses to sinusoidal were used for characterization of vestibular nuclei neurons as well as the study of population coding during artificial self-motion. Furthermore, at least 4 repetitions (i.e., trials) of naturalistic stimuli with a maximum head velocity of 200 deg/s and maximum acceleration of 14,700 deg/s^2^ in each direction was delivered. Naturalistic stimuli used here are a 60 s snippet of a recording of horizontal angular velocity from naturally behaving rhesus macaques [47,48].

We note that the maximum amplitude of the naturalistic stimulus was higher than that of the artificial sinusoidal stimuli irrespective of frequency. However, the actual amplitude of each frequency component of the naturalistic self-motion stimulus was lower than that of the artificial sinusoids (i.e., 15 deg/s; compare lower panels of Fig 1C and 1D). As such, the effective signal-to-noise ratio for each frequency is then expected to be lower during naturalistic as compared to during artificial self-motion stimulation, which would then give rise to lower coherence values overall as observed (e.g., compare Figs 2C and 3C).

### Spike sorting

All of the data presented in this paper consisted of well-isolated single units that were recorded on non-overlapping sites of the probe. As such, unit activities were sorted using MATLAB custom-made code as previously done for single neurons recording [44,51]. Specifically, we used voltage amplitude thresholding and visual inspection to detect the spiking activity from the background noise activity.

To assess the quality of sorted spikes and to evaluate the occurrence of type I and type II errors, involving incorrectly including noise spikes (those not belonging to a given neuron’s cluster) and excluding spikes from the given neuron’s cluster, respectively, we quantified the L_ratio_ and isolation distance for all the neurons [103]. This is important as including noise spikes or excluding spikes from a given neuron can affect the computation of the signal and noise correlation coefficients. L_ratio_ quantifies how well the spike cluster of a given neurons is separated from the spike cluster of other neurons or noise events [104]. Briefly, we calculated the energy of the spikes which is the sum of squared voltage values of the spikes over the interval from 1 ms before to 1.3 ms after the peak (please refer to [103,104] for complete description).

Spikes from a given cluster were normalized by their respective energy and formed a matrix with rows indicating spike waveform samples, and columns indicating the individual spike waveforms. The first principal component (PC) along with the energy of the spike for each neuron was used to form a feature matrix, containing energy and first PC vectors of all the neurons. The Mahalanobis distances of each spike, i, from a given cluster C was $D_{i,C}^{2}={\left(x_{i}-\mu_{C}\right)}^{T}\Sigma_{C}^{-1}(x_{i}-\mu_{C})$, where x_i_, μ_c_, and Σ_C_ are the feature vector, average of vectors in cluster C, and inverse covariance matrix of the vectors in cluster C. The value of L for cluster C was calculated as $L(C)=\sum_{i\notin C}(1-{CDF}_{\chi_{df}^{2}}(D_{i,C}^{2}))$, in which ${CDF}_{\chi_{df}^{2}}$ is the cumulative function of the χ^2^ distribution with df = 2N, where *N* is the number of neurons simultaneously recorded in a given session. Note that only spikes not belonging to cluster C were used in this calculation. The value of L(C) was then normalized by the number of spikes in cluster C to obtain L_ratio_ for that cluster.

Low values of L_ratio_ are associated with better sorted spikes and values less than 0.05 are associated with well-separated clusters and lesser type I and type II errors [103]. We note that all the L_ratio_ values in our dataset were all lower than 0.05 (S12A, S12B, and S12G Fig), indicating robust isolation of neurons and high fidelity in sorting of the spikes. The isolation estimates how distant the cluster spikes are from the other cluster spikes and noise [105]. If the cluster has n_C_ spikes, the isolation is the distance of the n_c_-th closest noise spike $j_{n_{C}},D_{j_{n_{C}},C}^{2}$. Higher isolation distance values correspond to lower type I errors [103] and clusters with isolation values exceeding 50 are deemed as effectively separated [105]. We note that the isolation values computed over our dataset were all above 100 (S12C–S12E Fig).

Finally, we quantified isolation using other standard measures such refractory period violations (i.e., the % of inter-spike intervals whose value was lesser or equal than 2 ms) and cluster overlap. Overall, refractory period violations were very low (<0.4% in all cases) (S12F Fig). Clusters for simultaneously recorded neuron pairs displayed minimal overlap (<4% in all cases; S12H Fig). Together, these results indicate very good if not excellent isolation of simultaneously recorded single units.

### Data analysis

Data was imported into MATLAB (MathWorks, Natick, Massachusetts, USA) programming environment for analysis. Binary sequences of unit activities were generated by setting the sequences at a given time to 1 if a spike happened at that time, and to 0, otherwise, and sampled at 1,000 Hz using custom-written codes for replaying neuronal recordings and sorting action potentials. The head position signal was calculated by integrating the head velocity signal. The eye position signal was computed as the difference between gaze and head position signals. The firing rate of the neurons was estimated by applying an optimal lowpass Kaiser filter to unit activities [106]. A neuron was categorized as a vestibular-only neuron if its response to pWBR stimulus did not depend on the eye movement (saccade as well as smooth pursuit), and if the response to VOR and VORc were identical.

Each neuron was also characterized as either type I or type II if it displayed increased firing rate in response to head movements towards the ipsilateral or contralateral sides, respectively. In practice, this was quantified by computing the phase between the firing rate and a sinusoidal head velocity stimulus at 1 Hz (see below). Neurons whose phases were near 0 were classified as type I, whereas neurons whose phases were near 180° were classified as type II.

### Linear systems analysis

Neural gain and phase values were computed for vestibular nuclei neurons in response to sinusoidal stimuli using traditional system identification techniques. We assumed that vestibular nuclei neurons responded to a sinusoidal stimulus, s(t), as fr(t) = g.s(t-t_d_) + b, where fr(t) is the estimated firing rate of the neuron; g is the neural gain; t_d_ is latency, the time by which the response of the neurons is leading the stimulus; and b is the bias. Fr(t) was estimated by lowpass filtering the unit activities with a cut-off frequency that exceeded the frequency of the stimulus by 0.1 Hz [106]. The response of the neuron across at least 10 trials were used to estimate b, t_d_, and g. t_d_ was estimated as the time at which the cross correlation of s(t) and fr(t) had maximum absolute value; next, g and b were obtained by performing a linear regression between fr(t) and s(t-t_d_). The response phase lead, p, was obtained as p = 360°t_d_f, where f is the frequency of the stimulus. To calculate the gain and phase during naturalistic stimulation, we computed $G(f)=\frac{P_{sr}(f)}{P_{ss}(f)}$, where P_sr_(f) is the cross power spectral density between stimulus and firing rate (i.e., the Fourier transform of the cross-correlation function between stimulus and firing rate), and P_ss_(f) is the power spectral density of the stimulus (i.e., the Fourier transform of the stimulus autocorrelation function). The gain and phase of the neuron in response to naturalistic stimulus were characterized as |G(f)|and ∠G(f) which denote magnitude and phase of the complex number G(f), respectively. The gain gives the number by which one needs to multiply the component of the stimulus at frequency f to get the corresponding component of the neural response. In contrast, the phase gives the number by which one must shift the stimulus component at frequency f to align with the corresponding component of the neural response. We note that G(f) is the transfer function between the stimulus and the neural response, which is then the number by which the Fourier transform of the stimulus must be multiplied to obtain the Fourier transform of the neural response. We further note that multiplication in the frequency domain corresponds to a convolution in the time domain, which is the most general linear transformation. For the case of a sinewave, convolution reduces to multiplication by a constant (i.e., the gain) and shifting by a time corresponding to the phase multiplied by the cycle period. Thus, the gain and phase values computed during naturalistic stimulation are a generalization of those computed for single sinewaves.

VO neural response properties to both artificial and naturalistic stimulation have been extensively studied. In particular, these neurons display similar high-pass dynamics to both naturalistic and artificial stimulation with gain and phase lead increasing as a function of frequency [43,45,51] (see [36,49] for review). We thus quantified the responses of single VO neurons to naturalistic and sinusoidal stimulation by computing the gain and phase as a function of frequency (S1 Fig). Overall, our results were in good agreement with those published previously (e.g., compare S1A and S1C Fig to Fig 3F and 3G of ref. [51], respectively for naturalistic; compare S1B and S1D to Fig 2B of ref. [36] for sinewaves). We next tested whether models based on gain and phase could predict responses to naturalistic and artificial stimuli for single neurons. The model was fitted to the first 50% of the data (i.e., gain and phase were computed as described above) and was tested on the remaining 50% of the data. In the case of naturalistic stimulation, a static sigmoidal nonlinearity was added to account for rectification and saturation as done previously [51]. Overall, the model accurately predicted responses to both naturalistic and artificial stimuli as quantified by R^2^, which is the correlation coefficient squared between the predicted and actual firing rate responses (S3 Fig).

### Quantification of heterogeneity

We quantified the heterogeneity of response of neurons across trials and across populations by calculating the response–response coherence of the neurons. This is because the response–response coherence is only limited by the variability of responses [53]. This measure ranges between 0 (i.e., both responses are uncorrelated) and 1 (i.e., both responses are equal up to a given time delay and/or proportionality constant). To compute the response–response coherence for a given neuron across trials (i.e., repetitions of the stimulus), we used $C_{RR}(f)=\frac{{\left|<P_{R_{i}R_{j}}(f){>}_{i\neq j}\right|}^{2}}{{\left(<P_{R_{i}R_{i}}(f)>\right)}^{2}}$, where R_i_ and R_j_ is the response of the neuron in trials i and j. Here, the average is performed over stimulus trials, such that $\langle \ldots \rangle =\frac{1}{N}\sum_{i=1}^{N}\ldots$, where *N* is the number of trials (i.e., the number of stimulus repetitions). We have ${\langle \ldots \rangle }_{i\neq j}=\frac{2}{N(N-1)}\sum_{i=2}^{N}\sum_{j=1}^{i-1}\ldots$. The response–response coherence of 2 neurons was calculated as $C_{RR}(f)=\frac{{\left|<P_{R_{i}R_{j}}(f)>\right|}^{2}}{<P_{R_{i}R_{i}}(f)><P_{R_{j}R_{j}}(f)>}$, where R_i_ is the responses of the first neuron in trial i and R_j_ is the response of the second neuron in trial j.

All spectral quantities (i.e., power-spectra, cross-spectra) were estimated using a multitaper technique with 8 Slepian functions [107] as done previously [43]. Heterogeneity was quantified as 1-C_RR_. This is because, as mentioned above, a response–response coherence of 1 would imply that responses are essentially similar (i.e., no heterogeneity), whereas a response–response coherence of 0 would imply that responses are dissimilar as they are uncorrelated (i.e., heterogeneous). Values were averaged over trials (4–5) for both naturalistic and artificial stimulation. In the case of artificial stimulation, consecutive stimulus cycles were concatenated to form a “trial” such that the number is equal to that of naturalistic stimulation. When computing the heterogeneity during naturalistic stimulation, we averaged the heterogeneity values over 0 to 5 Hz since the stimulus power primarily dominated in the frequency range. For artificial stimuli, we adopted the heterogeneity value at the stimulus frequency (i.e., averaged the heterogeneity over a 1 Hz window centered at the stimulus frequency).

### Correlation analysis

The correlation between the activities of a pair of neurons was quantified by computing spike count correlations [26]. To compute spike count correlations, a time window with given length (i.e., “timescale”) was used to compute spike counts from each neuron’s activity. The sequences of spike counts were generated for each neuron by counting the spikes within the temporal window. This procedure produced a pair of spike count sequences for a given pair. To eliminate the effect of noise correlations arising from the simultaneous recordings, we randomly shuffled the spike count sequences such that no spike count sequence of the neuron corresponded to the spike count sequence of the other neuron in the same trial (i.e., the so-called “shuffle predictor”) [54]. Signal correlations were then computed by calculating the Pearson’s correlation coefficients between these 2 spike count sequences (after shuffling).

For computing the noise correlations, residual spike count sequences were calculated by subtracting the original spike counts in a given trial from their average across trials for all trials. Noise correlations were computed by calculating Pearson’s correlation coefficients of the residual spike counts obtained in successive time windows with 50% overlap. We note that for calculation of signal correlations, we used both simultaneous and non-simultaneous pair of neurons, whereas for noise correlations, only simultaneously recorded pairs of neurons were considered. Values were averaged over trials (4–5) for both naturalistic and artificial stimulation. In the case of artificial stimulation, consecutive stimulus cycles were concatenated to form a “trial” such that the number is equal to that of naturalistic stimulation.

### Model

We simulated a population of vestibular nuclei neurons using linear-nonlinear cascade models [50,108]. The firing rate response of the neuron is given by $r(t)=T(r_{lin}(t))$ in which r_lin_(t) is the linear estimation neural response, and T(∙) is the nonlinear relation that relates r_lin_(t) to actual firing rate calculated from data. We note that we did not estimate the nonlinear function explicitly as this function was merged into an LIF model and its effect is reflected in parameters of LIF model. Parameters a, b, and c relate to maximum firing rate and neural sensitivity. $r_{lin}(t)=h(t)*s(t)+r_{0}$ in which h(∙) is the linear kernel of the model and the impulse response of the transfer function $H(f)=\frac{P_{sr}(f)}{P_{ss}(f)}$, where P_ss_(f) is the power spectrum of the stimulus and P_sr_(f) is the cross-spectrum between stimulus and binary sequence. H(f) was approximated with a transfer function similar to that of canal afferents with 2 poles and 2 zeros for each neuron as $H(s)=\frac{ks(s+1/T_{1})}{\left(s+1/T_{c})(s+1/T_{2}\right)},$ where s = 2πif and k, T_c_, T_1_, and T_2_ are parameters of the model [50]. r_0_ is the baseline firing rate which was calculated during baseline activity for each neuron and s(t) denotes the stimulus.

Power spectra and cross-spectrum quantities are computed using pwelch and cpsd functions in MATLAB. In practice, since we used inputs with naturalistic statistics, we fit the transfer function to the population averaged bode plot obtained for gain and phase during naturalistic stimuli. For controlled simulations, we kept the transfer functions similar across the population and used the following parameters: T_1_ = 0.0175 s; T_2_ = 0.027 s; T_c_ = 5.7 s; k = 2.1 (spk/s)/(deg/s).

To generate spiking activity, we fed the output of the linear transfer function to an LIF model [109]. The membrane potential, V(t), of the simulated neurons is calculated by solving $C_{m}\frac{dV(t)}{dt}=-gV(t)+I_{bias}+ks_{c}(t)+\sigma n_{c}(t)$, if $V(t_{0})\geq \theta \to V(t)=0(t_{0}\leq t\leq t+t_{ref})$. C_m_, g, I_bias_, k, σ, θ, and t_ref_ are membrane capacitance, membrane conductance, the bias current, input gain in the model, standard deviation of the noise, spike threshold, and refractory period, respectively. s_c_(t) denotes the input to the LIF model and **n**_**c**_(t) are the correlated noises which are defined below.

We obtained the distribution of signal and noise correlation coefficients and populated positive definite covariance matrices accordingly. We approximated the distribution of signal and noise correlations coefficients with normal distribution and populated the covariance matrices in a way that the distribution of the correlation coefficients followed the normal distribution. For homogenous population activity, we assumed σ = 0.03, whereas for heterogenous population activity, we assumed σ = 0.15, where σ is the standard deviation of the population. For populating large correlation matrices with values drawn from normal distribution with a given standard deviation (i.e., heterogeneity versus homogeneity) and mean value (mean value of signal and noise correlations), we used an algorithm based on vines and extended onion method [110]. We used this method as populating the off-diagonal elements of the covariance matrices with values randomly driven from an intended normal distribution resulted in non-positive definite covariance matrices for large populations (e.g., N>10 neurons). Specifically, **s**_*c*_(t) = L_sig_ **s**(t), where $s(t)=[s_{1}(t),\ldots ,s_{N}(t)]$ is the output of the transfer function, H(f), and L_sig_ is the Cholesky factor of the signal covariance matrix. Moreover, $n(t)=L_{n}{\left[\xi_{1}(t)\ldots \xi_{n}(t)\right]}^{T}$—where L_n_ is Cholesky decomposition of the noise covariance matrix and ξ_i_(t) for i = 1, …, N are white gaussian noise with mean zero and unit variance. For our simulations, we used C_m_ = 1 nF, g = 0.4 μS, I_bias_ = 7.3 nA, k = 0.025 nA (deg/s)/(spk/s), σ = 2 nA, θ = 0, and t_ref_ = 2 ms with which the resting discharge, coefficient of variation, variability, neural gain and phase of the simulated neurons matched that of population average values.

The correlated signal input was used as the signal component of the LIF model, whereas the correlated noise input was used as the noise component of the LIF model. We generated covariance matrices so that we could vary the mean of the off-diagonal correlation coefficients as well as their standard deviations systematically. Higher mean values corresponded to higher levels of noise and signal correlations. For simulations during heterogeneous conditions, the standard deviation values were 3 to 4 times higher than that of homogenous populations.

Previous studies have shown that the slope of the relationship between noise and signal correlations (i.e., the “correlation slope”) can strongly influence information transmission [56,57]. Because the correlation slope was different during naturalistic and artificial stimulation, changes in the slope were included in the simulations. Specifically, if the correlation matrices used to simulate signal and noise correlation in the spiking activity were the same, the slope of the correlations would be 1. Note that mean signal and noise correlation could be adjusted by subtracting or adding the off-diagonal correlation coefficients by appropriate value while keeping the slope 1 and leaving the standard deviation of the correlation coefficient distribution unchanged.

To obtain the desired correlation slope observed in data during naturalistic and artificial head motion, we used a similar method as in [57]. Specifically, we first generated correlated noise and signal matrices, **n**_**c**_(t) and **s**_**c**_(t) mentioned above, with the correlation slope of 1, and then randomly shuffled a portion of noise or signal vectors where each vector corresponds to noise and signal input to a simulated neuron in the LIF model. This operation ensured the slope would decrease without changing the correlation coefficient distribution.

Shuffling all the vectors results in a slope of 0 on average, which is similar to that of during artificial head motion (S9 Fig). In contrast, shuffling half of the vectors results in correlation slope of 0.5 on average, which is similar to that of during naturalistic head motion (S9 Fig). The correlation slope evaluated by performing a linear least squares fit. The results shown in the main figures during heterogeneous conditions were obtained using a correlation slope value that matched that of the data during naturalistic stimulation, whereas those obtained during homogeneous conditions were obtained using a correlation slope value that matched that of the data during artificial stimulation (S9 Fig).

### Stimulus reconstruction and decoding

We used the stimulus reconstruction technique in order to estimate the stimulus waveform from the model neurons’ spiking activities [43,111]. The estimated stimulus is obtained by convolving each neural activity with a kernel: $s_{est}(t)=\sum_{i=1}^{N}\left(k_{i}*r_{i})(t\right)$. Here, r_i_(t) is the firing rate and k_i_(t) is the optimal kernel of the neuron i. The kernels are obtained from the following equation:

$$\left(\begin{array}{c} K_{1}(f)\\ \vdots \\ K_{N}(f) \end{array}\right)={(\begin{array}{ccc} P_{r_{1}r_{1}}(f) & \cdots & P_{r_{1}r_{N}}(f)\\ \vdots & \ddots & \vdots \\ P_{r_{N}r_{1}}(f) & \cdots & P_{r_{N}r_{N}}(f) \end{array})}^{-1}\left(\begin{array}{c} P_{sr_{1}}(-f)\\ \vdots \\ P_{sr_{N}}(-f) \end{array}\right),$$

where K_i_(f) is the Fourier transform of k_i_(t), $P_{r_{i}r_{j}}(f)$ is the cross-spectrum between r_i_(t) and r_j_(t), and $P_{sr_{i}}(f)$ is the cross-spectrum stimulus, s(t), and r_i_(t).n(t) = s_est_(t)−s(t) is the noise in the reconstruction and mean squared error (MSE) is defined as ε^2^ = <n^2^(t)>. Signal-to-noise ratio (SNR) is computed via $SNR(f)=\frac{P_{ss}(f)}{P_{nn}(f)}$ in which P_ss_(f) and P_nn_(f) are the power spectrum of stimulus and reconstruction noise, respectively. The MSE is given by: *ε*^2^ = 〈*n*^2^(*t*)〉, where 〈…〉 denotes an average over time. The mutual information rate is then given by [112]:

$$MI=\int_{0}^{20}df\;{log}_{2}[1+SNR(f)],$$

where the limits of integration correspond to the frequency range of natural self-motion [49]. Information rate values were normalized by the lowest value obtained, which was for a homogeneous population for zero mean signal and highest mean noise correlations (bottom right corner of Fig 5C). We used the coding fraction to assess the goodness-of-fit for a given stimulus estimation [43,111,112]. The coding fraction was computed as $1-\frac{\varepsilon }{\sigma }$, where σ is the standard deviation of the stimulus.

### Covariance analysis

To gain intuition as to how noise correlations benefit population coding for neuronal populations with heterogenous signal and noise correlation structures, we looked at the contributions of individual terms towards determining the mean square error. Specifically, one can write an expression for the MSE as:

$$\varepsilon^{2}=\langle {\left(\sum_{i=1}^{N}{err}_{i}\right)}^{2}\rangle =\sum_{i=1}^{N}\langle {err}_{i}^{2}\rangle +2\sum_{i=2}^{N}\sum_{j=1}^{i-1}\langle {err}_{i}{err}_{j}\rangle ,$$

where *N* is the population size and *err*_*i*_ is the contribution of neuron *i* to the error, which is given by ${err}_{i}=\frac{s(t)}{N}-(k_{i}*r_{i})(t)$. It can be easily seen that the equation above is then a sum of variance and covariance terms. We fit the MSE as a function of population size curves with 2 different power law functions over different ranges [47]. The critical population size was defined as the value of population size for which both power laws intersect.

## Supporting information

S1 Fig Neural gains for naturalistic (A) and artificial (B) stimulation as well as phases for naturalistic (C) and artificial (D) stimulation. For each panel, curves from individual neurons are shown in gray. A few individual neurons are shown in color (cyan, yellow, magenta, green). The population-averages traces during naturalistic and artificial stimulation are show by thick red and blue lines, respectively. Shaded areas and error bars indicate 1 SEM. *N* = 41, naturalistic stimulus; *N* = 42, f = 0.5 HZ; *N* = 42, f = 1 HZ; *N* = 40, f = 2 HZ; *N* = 41, f = 3 HZ; *N* = 40, f = 4 HZ; *N* = 41, f = 5 HZ; *N* = 39, f = 8 HZ; *N* = 37, f = 17 HZ.
(TIF)

S2 Fig Neural gains for naturalistic (A) and artificial (B) stimulation as well as phases for naturalistic (C) and artificial (D) stimulation. Population-averaged curves are plotted for each animal. The shaded areas demonstrate 1 SEM. Monkey O, *N* = 4; Monkey B, *N* = 34; Monkey D, *N* = 11. The bands show 1 SEM.
(TIF)

S3 Fig *Top*: Example naturalistic (A) and artificial (B) sinusoidal head velocity stimulus (f = 4 Hz) used in the study. (C and D) Time-dependent firing rates for 3 exemplar type I and II neurons (gray band) as well as predicted firing rate from the best-fit model (black curves). The insets in A and B shows the goodness-of-fit of the models during naturalistic (*N* = 41) and artificial (*N* = 322) stimulation, respectively. Note that the model was fit to 50% of the data and the goodness-of-fit quantified on the remaining 50%.
(TIFF)

S4 Fig Neural activity is heterogenous in response to sinusoidal stimulation with differential frequency. The population-averaged response–response coherence, as well as the corresponding heterogeneity (inset), is shown across trials (blue) and neurons (magenta) for stimulation sinusoidal stimuli with the frequency of 0.5 Hz (*N* = 42 neurons; S4A Fig), 1 Hz (*N* = 42 neurons; S4B Fig), 2 Hz (*N* = 40 neurons; S4C Fig), 3 Hz (*N* = 41 neurons; S4D Fig), 5 Hz (*N* = 41 neurons; S4E Fig), 8 Hz (*N* = 39 neurons; S4F Fig), and 17 Hz (*N* = 0.37 neurons; S4G Fig). Across all frequencies, the contribution of trial-to-trail variability and the variability across neurons to heterogeneity was not significantly different from each other (Wilcoxon rank sum test, insets: 0.5 Hz, *p* = 0.86, *N* = 42; 1 Hz, *p* = 0.59, *N* = 42; 2 Hz, *p* = 0.62, *N* = 40; 3 Hz, *p* = 0.23, *N* = 41; 5 Hz, *p* = 0.25, *N* = 41; 8 Hz, *p* = 0.39, *N* = 39; S1G, *p* = 0.49, *N* = 37). The bands show 1 SEM. The data for all panels are available from the Borealis database (https://doi.org/10.5683/SP3/FXFZ2J) (see files “FigS4X.mat,” “FigS4X.m,” and associated “readme.txt,” where “X” corresponds to the panel letter).
(TIF)

S5 Fig Signal correlation as a function of timescale during sinusoidal stimulation with different frequencies: S5A Fig: 0.5 Hz, *N* = 861 pairs; S5B: 1 Hz, *N* = 861 pairs; S5C: 2 Hz, *N* = 780 pairs; S5D: 3 Hz, *N* = 820 pairs; S5E: 5 Hz, *N* = 820 pairs; S5F: 8 Hz, *N* = 741 pairs; and S5G: 17 Hz, *N* = 666 pairs. The blue and purple lines in each panel represent the correlations for the same-type and opposite-type pairs, respectively. The thick solid blue and dashed purple lines are the average values of the correlations for the same-type and opposite-type pairs, respectively. While the average values were calculated using all the pairs, only 75 traces of same and opposite-type pairs were shown for visualization purposes.
(TIF)

S6 Fig Noise correlation as a function of timescale during sinusoidal stimulation with different frequencies: S6A Fig, *N* = 28 pairs: 0.5 Hz; S6B: 1 Hz, *N* = 29 pairs; S6C: 2 Hz, *N* = 35 pairs; S6D: 3 Hz, *N* = 35 pairs; S6E: 5 Hz, *N* = 34 pairs; S6F: 8 Hz, *N* = 33 pairs; and S6G: 17 Hz, *N* = 33 pairs. The blue and purple lines in each panel represent the correlations for the same-type and opposite-type pairs, respectively. The thick solid blue and dashed purple lines are the average values of the correlations for the same-type and opposite-type pairs, respectively.
(TIF)

S7 Fig Signal **(A)** and noise **(B)** correlations during naturalistic stimulation when only using naturalistic stimulus segments for which amplitude is below the threshold. The bottom panels show the % of the naturalistic stimulus that were used for each threshold. Overall, no significant changes were seen when systematically varying the threshold (A: *p* > 0.76; B: *p* > 0.99 between naturalistic stimuli with different thresholds, *p* = 1.4 × 10^−13^ between naturalistic and artificial stimuli for noise correlations; Wilcoxon rank-sum tests). Signal and noise correlations are also shown during artificial stimulation for comparison (blue). The data for all panels are available from the Borealis database (https://doi.org/10.5683/SP3/FXFZ2J) (see files “FigS7X.mat,” “FigS7X.m,” and associated “readme.txt,” where “X” corresponds to the panel letter).
(TIF)

S8 Fig Firing rates during naturalistic (red) and artificial (blue) stimulation. No significant differences were observed (*p* > 0.64 in all cases, one-way ANOVA with Bonferroni correction). *N* = 41, naturalistic stimulus; *N* = 42, f = 0.5 HZ; *N* = 42, f = 1 HZ; *N* = 40, f = 2 HZ; *N* = 41, f = 3 HZ; *N* = 40, f = 4 HZ; *N* = 41, f = 5 HZ; *N* = 39, f = 8 HZ; *N* = 37, f = 17 HZ. The data for this figure are available from the Borealis database (https://doi.org/10.5683/SP3/FXFZ2J) (see files “FigS8.mat,” “FigS8.m,” and associated “readme.txt”).
(TIF)

S9 Fig Noise correlations as a function of signal correlations during naturalistic **(A)** and artificial **(B)** stimulation. There was a significant relationship between both during naturalistic (r = 0.50, *p* = 1.5 × 10^−78^; Pearson’s correlation coefficient) but not artificial stimulation (r = −0.01, *p* = 0.11; Pearson’s correlation coefficient). The dashed line shows the best-fit straight line (A: slope = 0.45, R^2^ = 0.26; B: slope = −0.01, R^2^ = 3.1 × 10^−4^).
(TIF)

S10 Fig **(A)** Information rate as a function of mean signal and mean noise correlations for the homogeneous case. For given values of mean signal and noise correlations, we assumed that signal and noise correlations were independent of one another as seen experimentally during artificial stimulation (S9B Fig). **(B)** Information rate as a function of mean signal and mean noise correlations for the heterogeneous case where signal and noise correlations are independent of one another (i.e., the correlation slope is zero). **(C)** Information rate as a function of mean signal and mean noise correlations for the heterogeneous case where the relationship between signal and noise correlations has the same slope as that seen experimentally during naturalistic stimulation (S9A Fig). **(D)** Information gain rate as function of mean noise correlation for heterogenous (ρ_signal_ = 0) and homogenous (green) conditions for mean signal correlation ρ_signal_ = 0.6. For heterogeneous conditions, we compared values obtained when signal and noise correlations were independent of one another (purple) to those obtained when signal and noise correlations were related as seen experimentally during naturalistic stimulation (orange). Error bands indicate 1 SEM (*N* = 40 simulations). It is seen that information rates were lower when signal and noise correlations were related, consistent with previous studies, but were still higher than those obtained in the homogeneous condition.
(TIF)

S11 Fig **(A)** Coding fraction as a function of mean signal and mean noise correlations for the homogeneous case. For given values of mean signal and noise correlations, we assumed that signal and noise correlations were independent of one another as seen experimentally during artificial stimulation (S9B Fig). **(B)** Coding fraction as a function of mean signal and mean noise correlations for the heterogeneous case where the relationship between signal and noise correlations has the same slope as that seen experimentally during naturalistic stimulation (S9A Fig). **(C)** Correlation coefficients as a function of mean signal and noise correlations for the homogenous conditions. As for the simulations in panel A, we assumed that signal and noise correlations were independent as seen experimentally. **(D)** Correlation coefficients as a function of mean signal and noise correlations for the heterogenous conditions. As for the simulations in panel B, we assumed that signal and noise correlations were correlated with the same slope as seen experimentally. As seen for the information rate (S10A Fig), coding fraction and correlation coefficient values increase with higher signal correlations and lower noise correlations when during homogenous condition. In contrast, as seen for the information rate (S10C Fig), coding fraction and correlation coefficient values increase with higher noise correlations and lower signal correlations when during heterogenous population activity.
(TIF)

S12 Fig **(A)** Schematic of the multi-channel probe and signals recorded on separate sites (14 in red and 32 in cyan, together with adjacent sites; schematic reproduced from manual for V1x32-Poly2-15mm-50s-177 manual from NeuroNexus website, https://neuronexus.com/). Also shown are extracellular spike waveforms aligned to time of occurrence (thin curves) and population averages (thick curves). **(B)** Plot in feature space of both units. The features in x-axis and y-axis are the energy of spikes and first principal component of feature matrix, respectively, as describe in methods section. Red and blue dots represent scatter plots for spikes of first and second units obtained from channels 14 and 24 on the probe, respectively. The L-ratio values for each neuron are indicated in the figure. **(C)** Scatter plot of spike clusters of the first unit versus noise in feature space. Energy of spikes in channels 1 and 4 are used for this plot. The isolation distance computed between cluster and noise spikes is 762 for this unit. **(D)** Scatter plot of spike clusters of the second unit versus noise in feature space. Energy of spikes in channels 1 and 3 are used for this plot. The isolation distance computed between cluster and noise spikes is 158 for this unit. In both panels C and D, a third of spikes are shown randomly for visualization purposes. **(E)** Histogram of isolation distance calculated for all units (*N* = 41). All values were above 100 indicating good if not excellent separation between cluster and noise spikes. **(F)** Histogram of refractory violation for single neurons in the population (*N* = 41). **(G)** L_ratio_ calculated for all the neurons in the population (*N* = 41). All values are less than 0.05. **(H)** Percentage overlap between clusters of neurons in pairs (similar to that in panel B; *N* = 35).
(TIF)

## Abbreviations

LIF: leaky integrate-and-fire
MSE: mean squared error
PC: principal component
PIVC: parietoinsular vestibular cortex
pWBR: passive whole-body rotation
SEM: Standard error of the mean
SNR: signal-to-noise ratio
VIP: ventral intraparietal
VN: vestibular nucleus
VO: vestibular-only
VOR: vestibulo-ocular reflex
VORc: vestibulo-ocular reflex cancellation
VPL: ventro posterior lateral
