# Supplementary figures and images for: Neural populations within macaque early vestibular pathways are adapted to encode natural self-motion
Source: PLoS Biol. 2024 Apr 30;22(4):e3002623. doi: 10.1371/journal.pbio.3002623 (PMC11086886; doi:10.1371/journal.pbio.3002623)

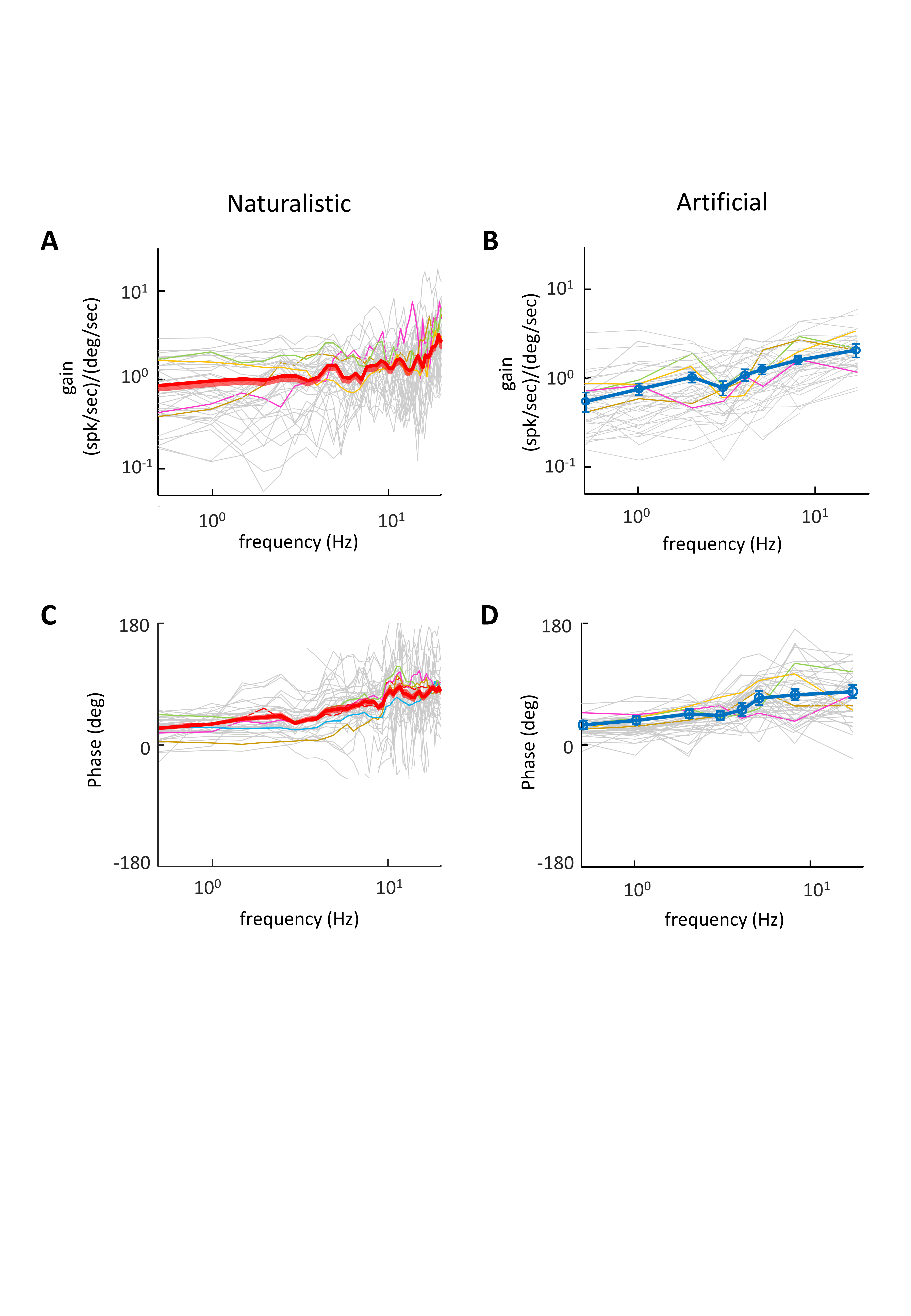

Supplement: S1 Fig — Neural gains for naturalistic (A) and artificial (B) stimulation as well as phases for naturalistic (C) and artificial (D) stimulation. For each panel, curves from individual neurons are shown in gray. A few individual neurons are shown in color (cyan, yellow, magenta, green). The population-averages traces during naturalistic and artificial stimulation are show by thick red and blue lines, respectively. Shaded areas and error bars indicate 1 SEM. N = 41, naturalistic stimulus; N = 42, f = 0.5 HZ; N = 42, f = 1 HZ; N = 40, f = 2 HZ; N = 41, f = 3 HZ; N = 40, f = 4 HZ; N = 41, f = 5 HZ; N = 39, f = 8 HZ; N = 37, f = 17 HZ. (TIF) [file pbio.3002623.s001.tif]

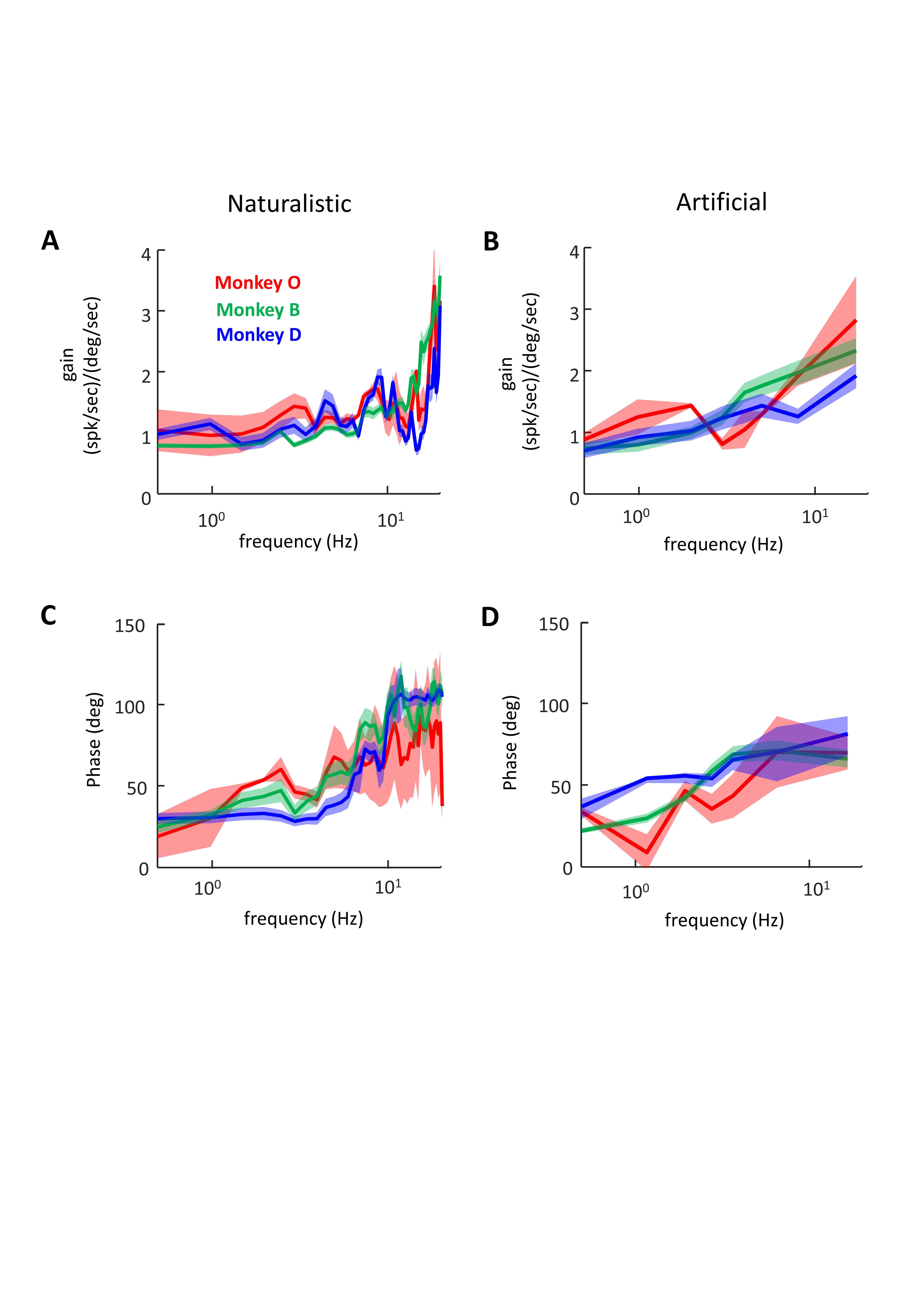

Supplement: S2 Fig — Neural gains for naturalistic (A) and artificial (B) stimulation as well as phases for naturalistic (C) and artificial (D) stimulation. Population-averaged curves are plotted for each animal. The shaded areas demonstrate 1 SEM. Monkey O, N = 4; Monkey B, N = 34; Monkey D, N = 11. The bands show 1 SEM. (TIF) [file pbio.3002623.s002.TIF]

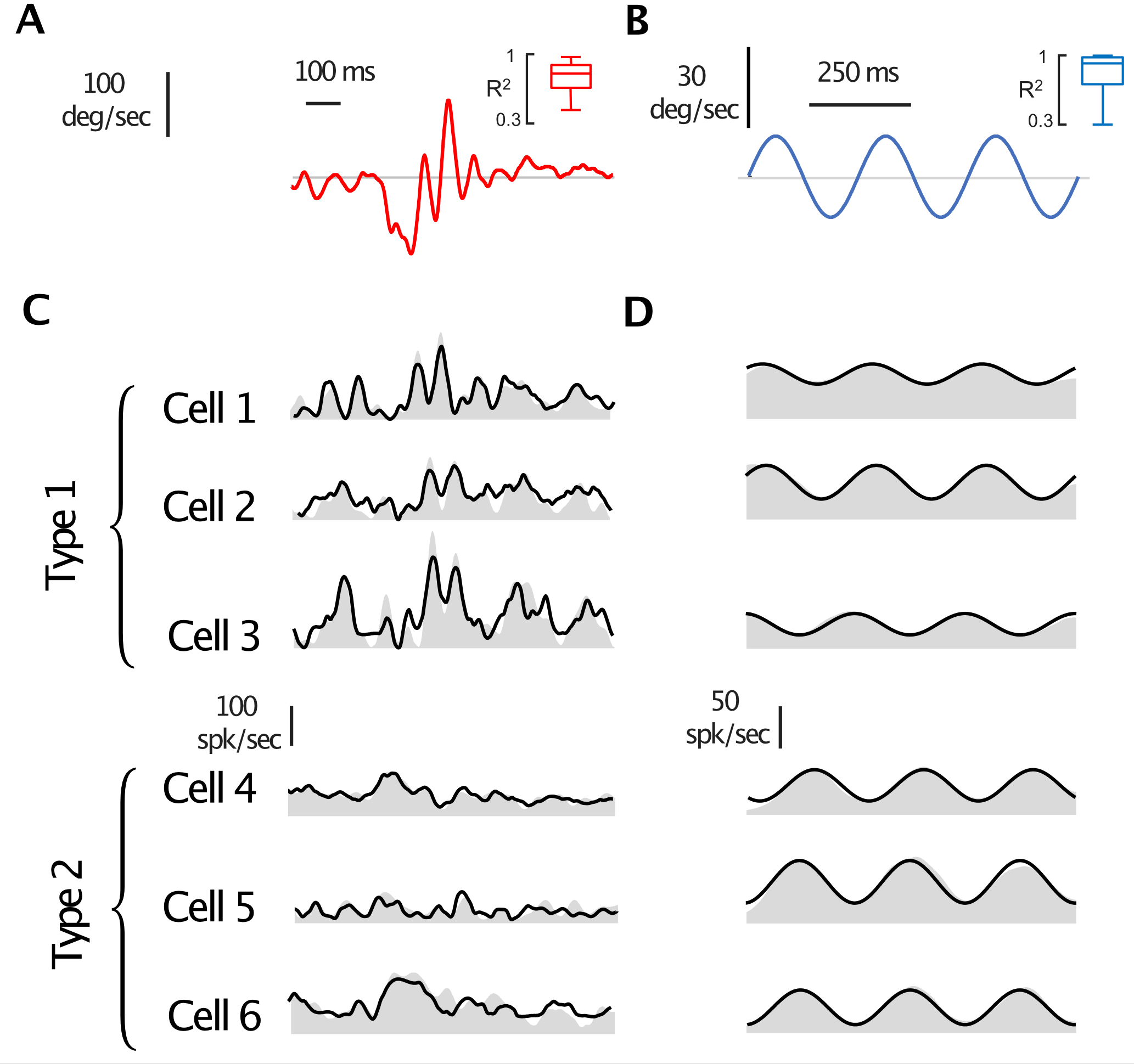

Supplement: S3 Fig — Top: Example naturalistic (A) and artificial (B) sinusoidal head velocity stimulus (f = 4 Hz) used in the study. (C and D) Time-dependent firing rates for 3 exemplar type I and II neurons (gray band) as well as predicted firing rate from the best-fit model (black curves). The insets in A and B shows the goodness-of-fit of the models during naturalistic (N = 41) and artificial (N = 322) stimulation, respectively. Note that the model was fit to 50% of the data and the goodness-of-fit quantified on the remaining 50%. (TIFF) [file pbio.3002623.s003.tiff]

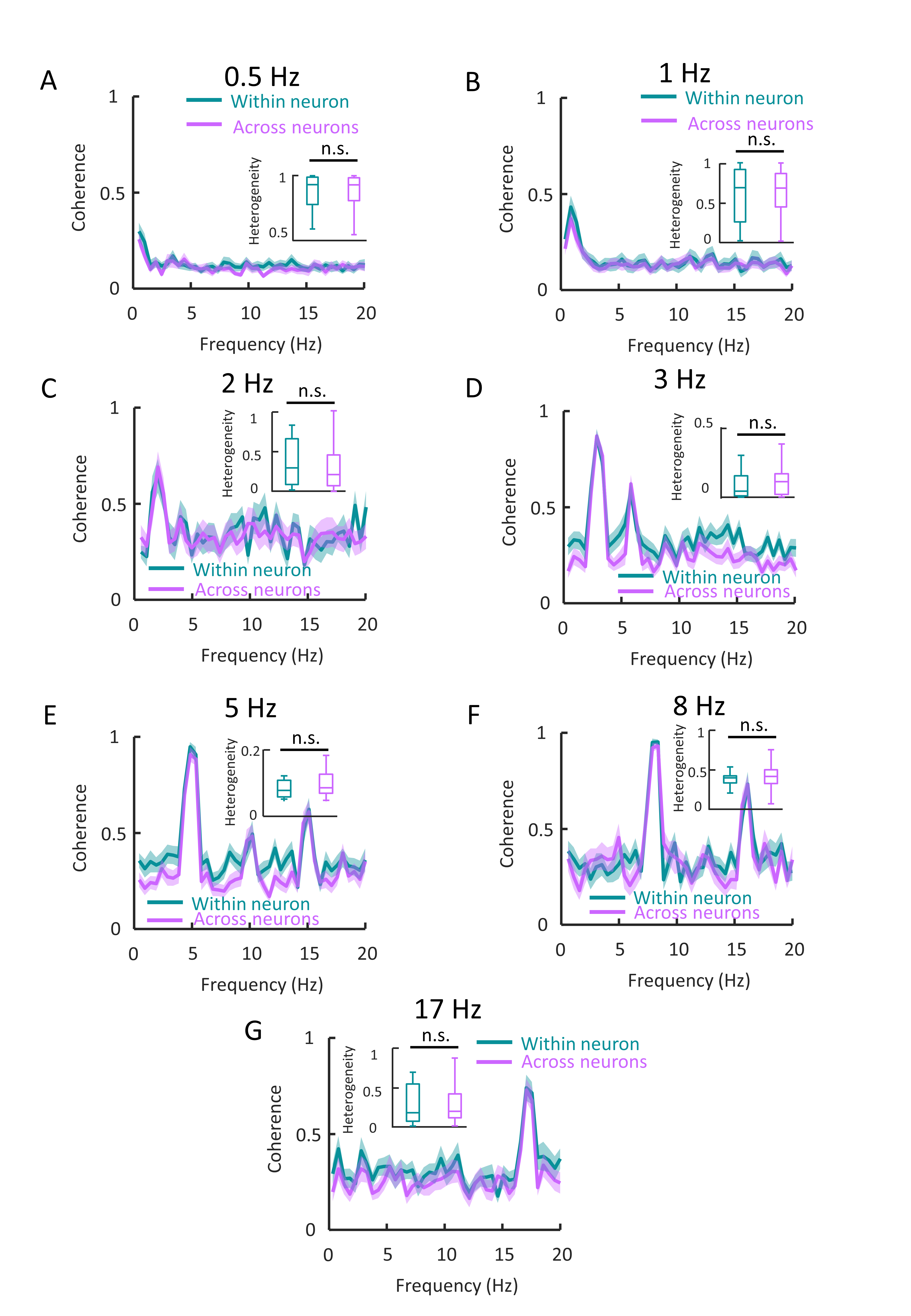

Supplement: S4 Fig — The population-averaged response–response coherence, as well as the corresponding heterogeneity (inset), is shown across trials (blue) and neurons (magenta) for stimulation sinusoidal stimuli with the frequency of 0.5 Hz (N = 42 neurons; S4A Fig), 1 Hz (N = 42 neurons; S4B Fig), 2 Hz (N = 40 neurons; S4C Fig), 3 Hz (N = 41 neurons; S4D Fig), 5 Hz (N = 41 neurons; S4E Fig), 8 Hz (N = 39 neurons; S4F Fig), and 17 Hz (N = 0.37 neurons; S4G Fig). Across all frequencies, the contribution of trial-to-trail variability and the variability across neurons to heterogeneity was not significantly different from each other (Wilcoxon rank sum test, insets: 0.5 Hz, p = 0.86, N = 42; 1 Hz, p = 0.59, N = 42; 2 Hz, p = 0.62, N = 40; 3 Hz, p = 0.23, N = 41; 5 Hz, p = 0.25, N = 41; 8 Hz, p = 0.39, N = 39; S1G, p = 0.49, N = 37). The bands show 1 SEM. The data for all panels are available from the Borealis database (https://doi.org/10.5683/SP3/FXFZ2J) (see files “FigS4X.mat,” “FigS4X.m,” and associated “readme.txt,” where “X” corresponds to the panel letter). (TIF) [file pbio.3002623.s004.TIF]

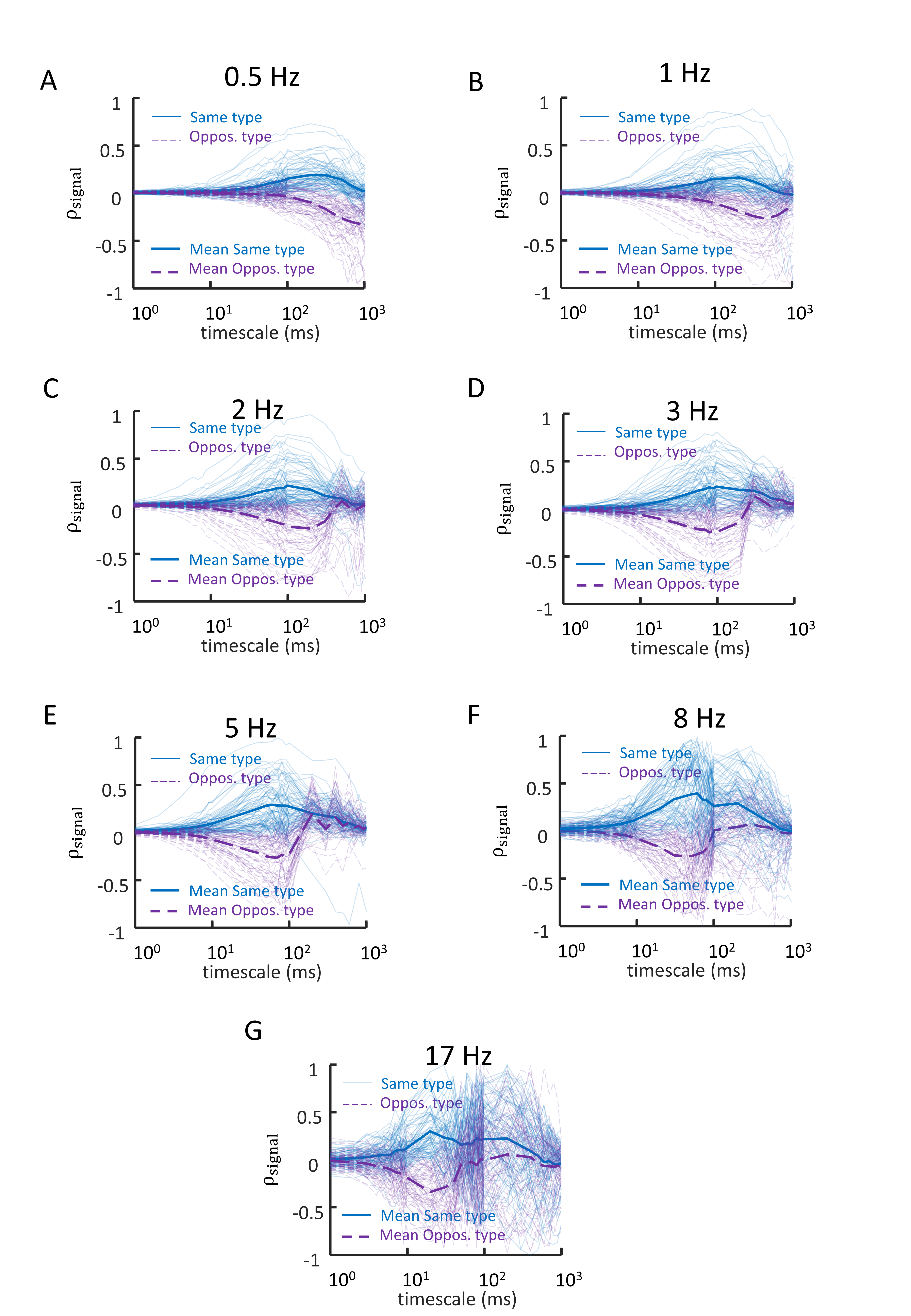

Supplement: S5 Fig — The blue and purple lines in each panel represent the correlations for the same-type and opposite-type pairs, respectively. The thick solid blue and dashed purple lines are the average values of the correlations for the same-type and opposite-type pairs, respectively. While the average values were calculated using all the pairs, only 75 traces of same and opposite-type pairs were shown for visualization purposes. (TIF) [file pbio.3002623.s005.TIF]

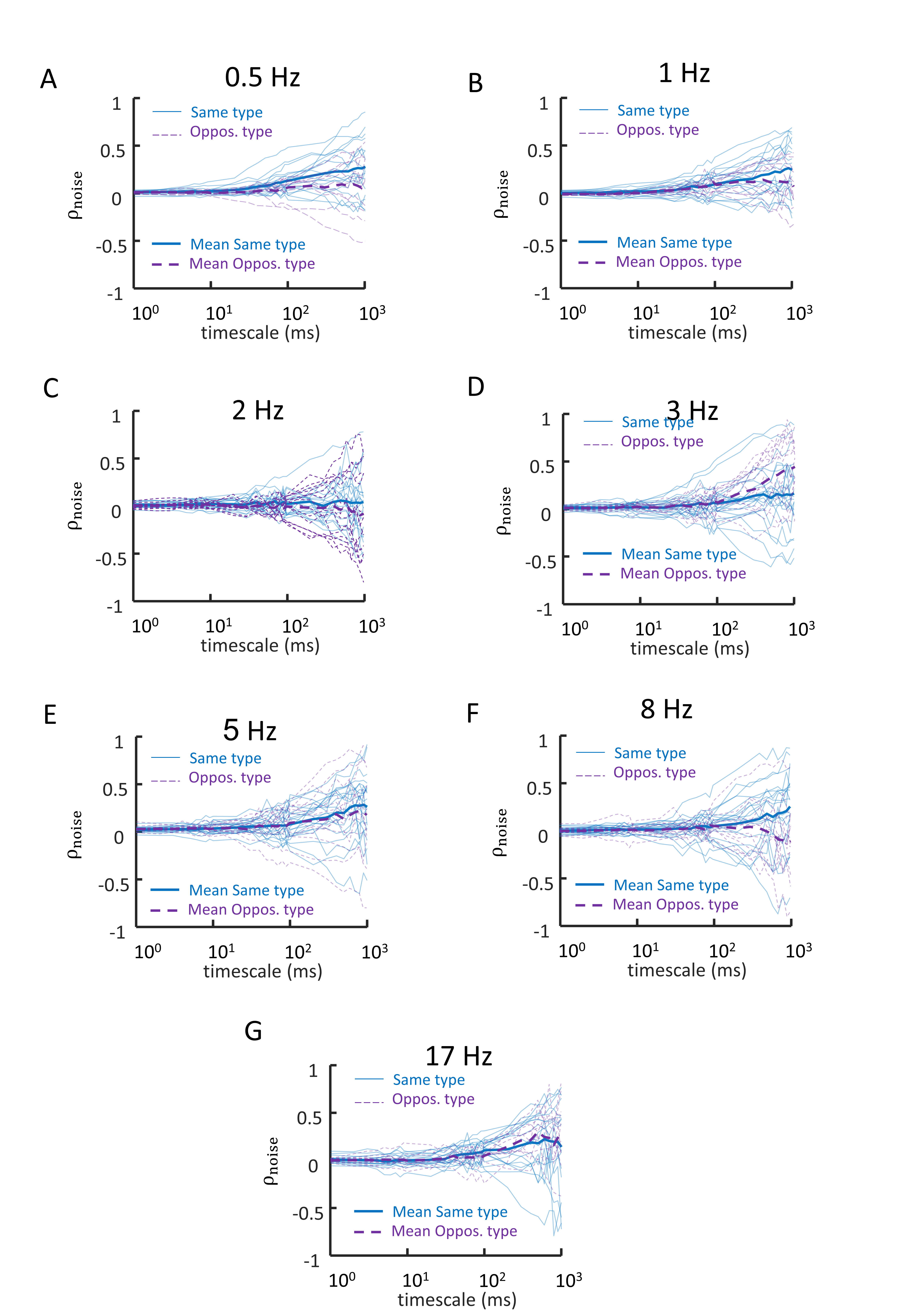

Supplement: S6 Fig — The blue and purple lines in each panel represent the correlations for the same-type and opposite-type pairs, respectively. The thick solid blue and dashed purple lines are the average values of the correlations for the same-type and opposite-type pairs, respectively. (TIF) [file pbio.3002623.s006.TIF]

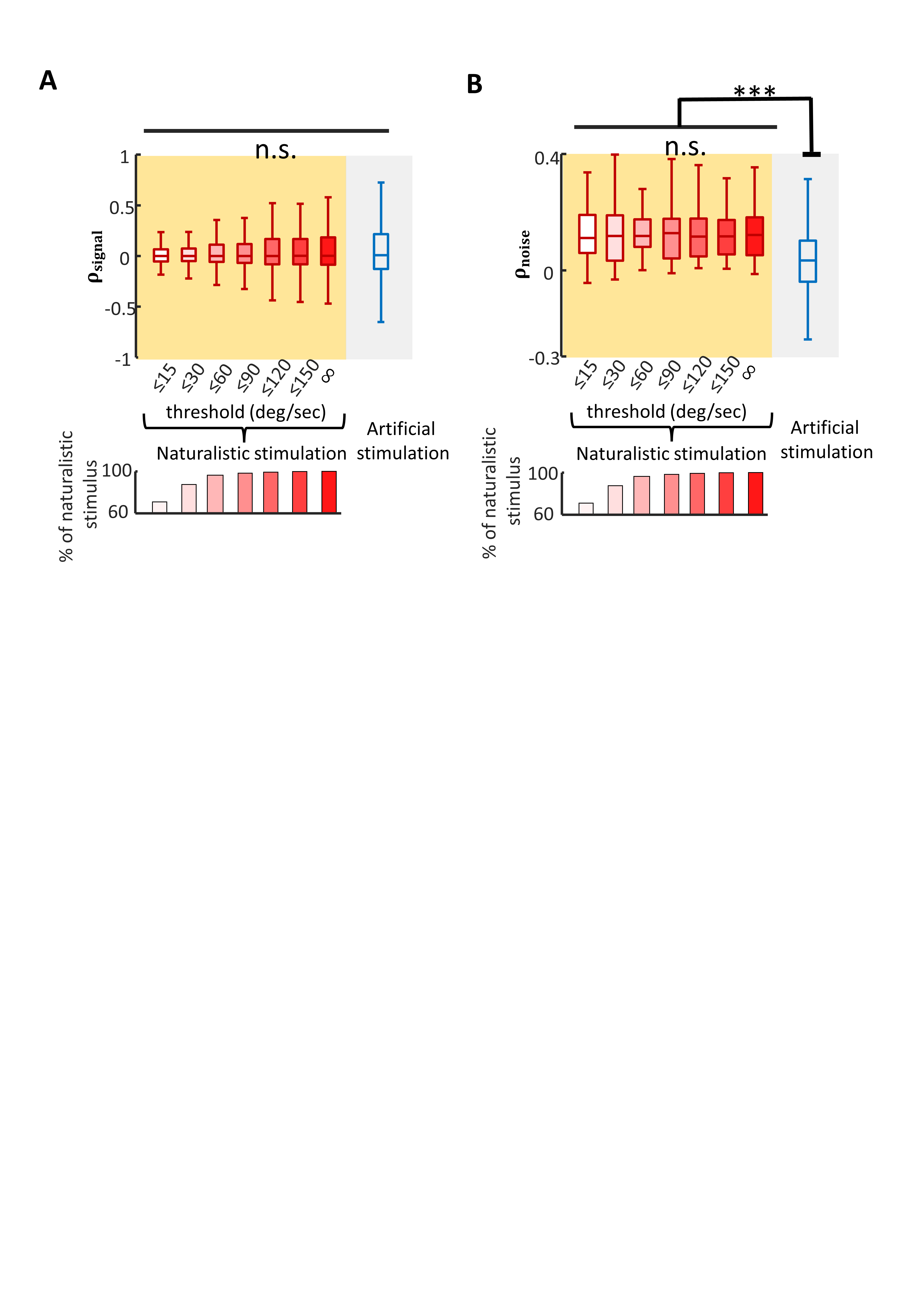

Supplement: S7 Fig — Signal (A) and noise (B) correlations during naturalistic stimulation when only using naturalistic stimulus segments for which amplitude is below the threshold. The bottom panels show the % of the naturalistic stimulus that were used for each threshold. Overall, no significant changes were seen when systematically varying the threshold (A: p > 0.76; B: p > 0.99 between naturalistic stimuli with different thresholds, p = 1.4 × 10^−13^ between naturalistic and artificial stimuli for noise correlations; Wilcoxon rank-sum tests). Signal and noise correlations are also shown during artificial stimulation for comparison (blue). The data for all panels are available from the Borealis database (https://doi.org/10.5683/SP3/FXFZ2J) (see files “FigS7X.mat,” “FigS7X.m,” and associated “readme.txt,” where “X” corresponds to the panel letter). (TIF) [file pbio.3002623.s007.TIF]

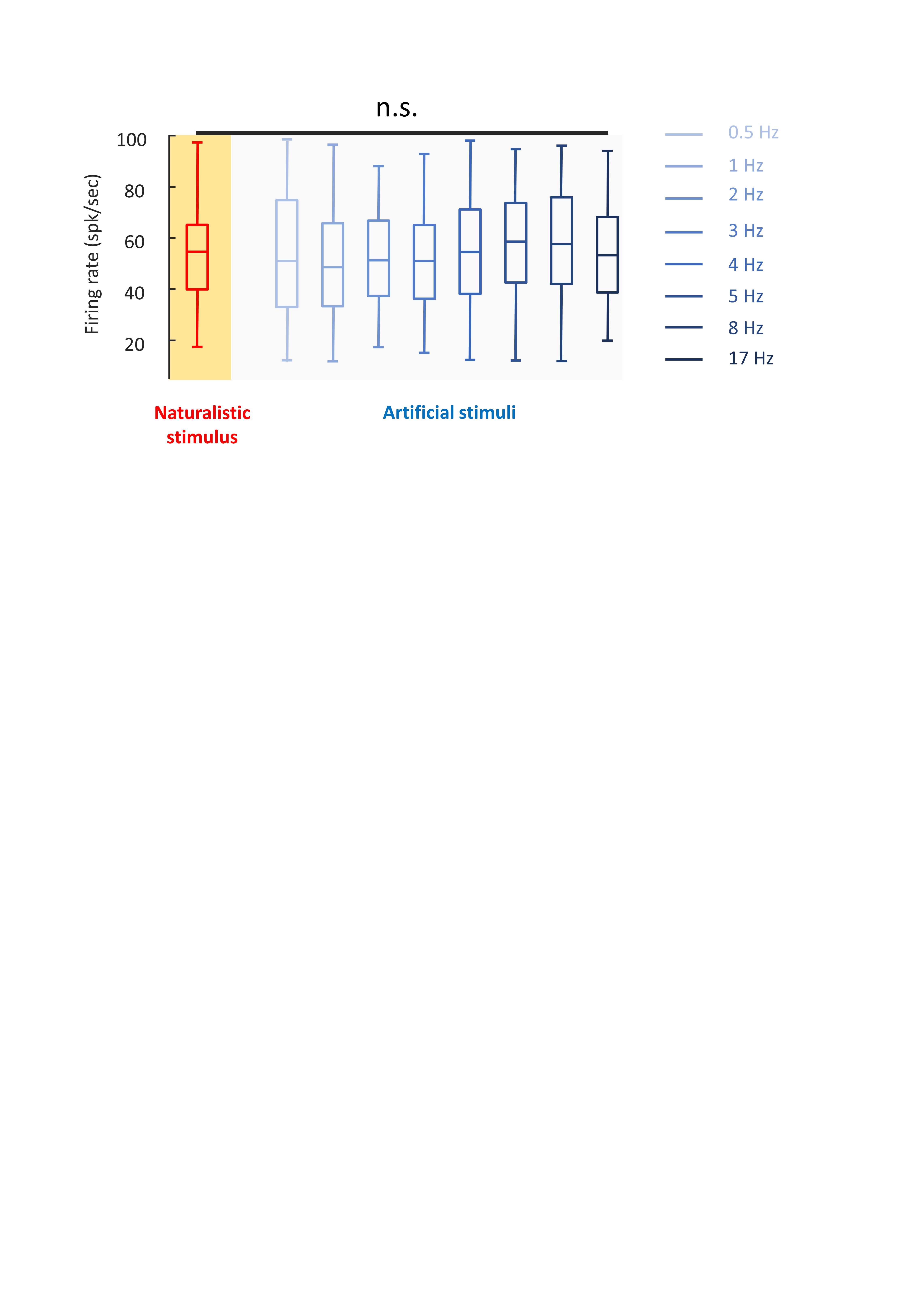

Supplement: S8 Fig — No significant differences were observed (p > 0.64 in all cases, one-way ANOVA with Bonferroni correction). N = 41, naturalistic stimulus; N = 42, f = 0.5 HZ; N = 42, f = 1 HZ; N = 40, f = 2 HZ; N = 41, f = 3 HZ; N = 40, f = 4 HZ; N = 41, f = 5 HZ; N = 39, f = 8 HZ; N = 37, f = 17 HZ. The data for this figure are available from the Borealis database (https://doi.org/10.5683/SP3/FXFZ2J) (see files “FigS8.mat,” “FigS8.m,” and associated “readme.txt”). (TIF) [file pbio.3002623.s008.TIF]

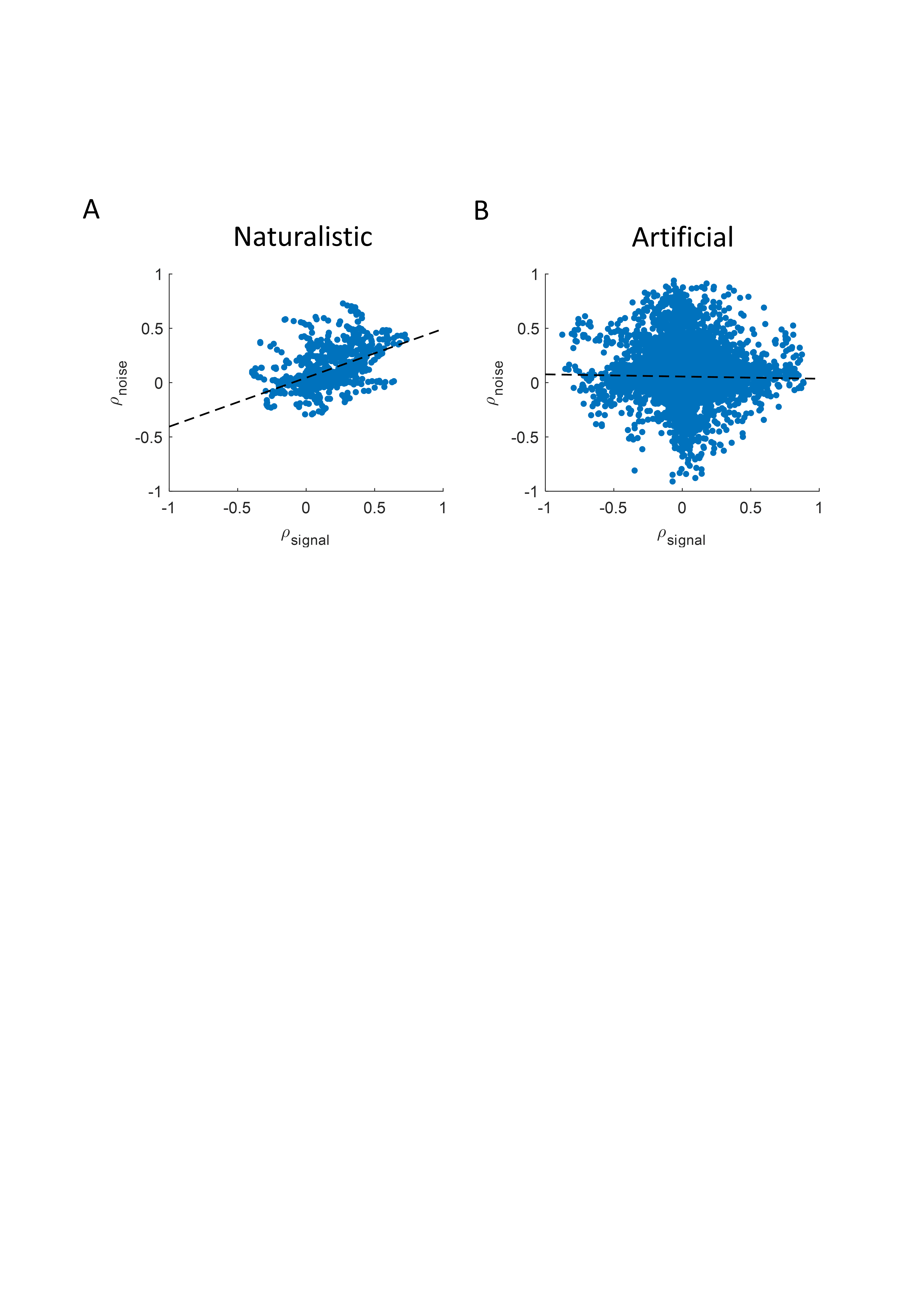

Supplement: S9 Fig — Noise correlations as a function of signal correlations during naturalistic (A) and artificial (B) stimulation. There was a significant relationship between both during naturalistic (r = 0.50, p = 1.5 × 10^−78^; Pearson’s correlation coefficient) but not artificial stimulation (r = −0.01, p = 0.11; Pearson’s correlation coefficient). The dashed line shows the best-fit straight line (A: slope = 0.45, R2 = 0.26; B: slope = −0.01, R2 = 3.1 × 10^−4^). (TIF) [file pbio.3002623.s009.TIF]

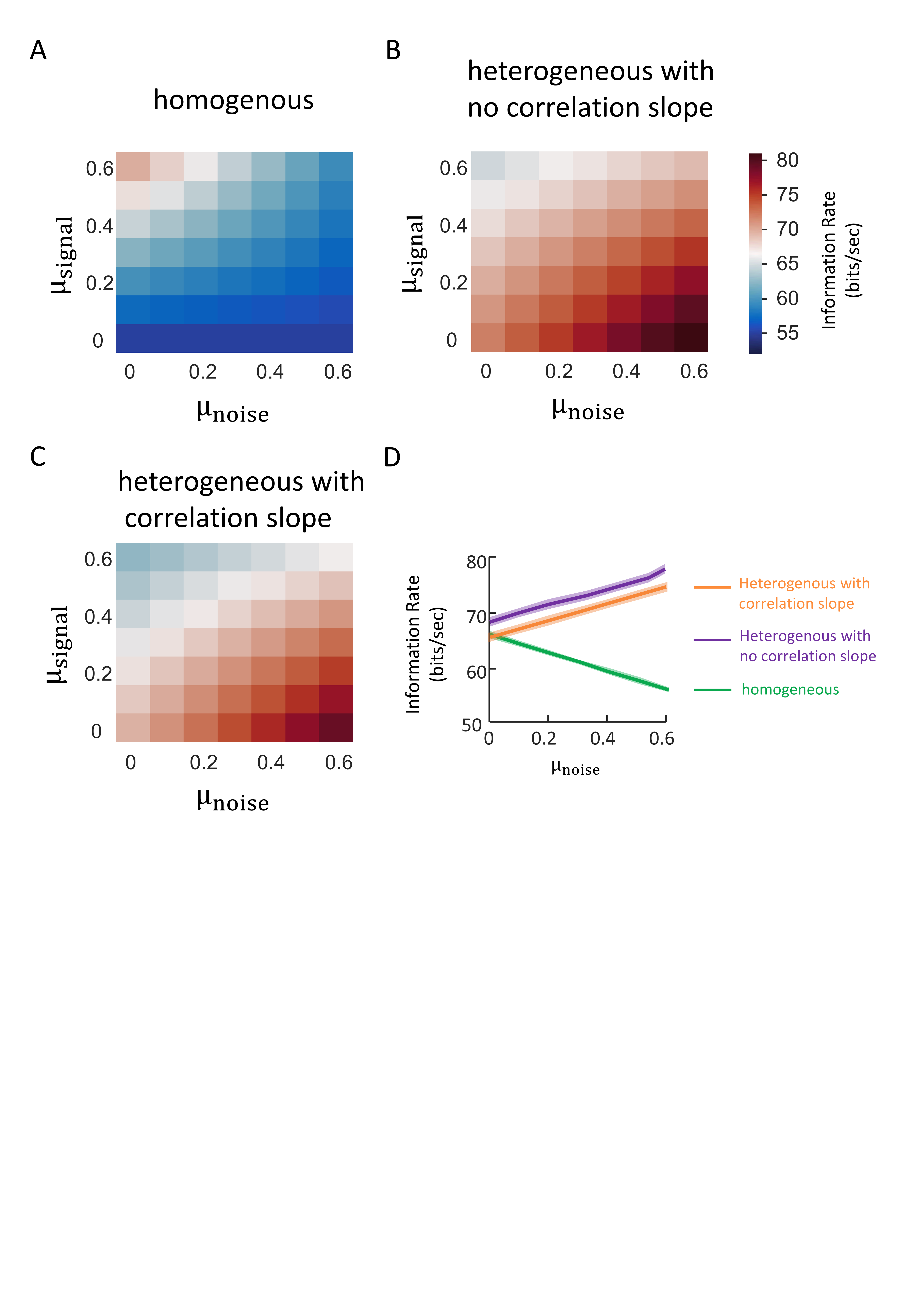

Supplement: S10 Fig — (A) Information rate as a function of mean signal and mean noise correlations for the homogeneous case. For given values of mean signal and noise correlations, we assumed that signal and noise correlations were independent of one another as seen experimentally during artificial stimulation (S9B Fig). (B) Information rate as a function of mean signal and mean noise correlations for the heterogeneous case where signal and noise correlations are independent of one another (i.e., the correlation slope is zero). (C) Information rate as a function of mean signal and mean noise correlations for the heterogeneous case where the relationship between signal and noise correlations has the same slope as that seen experimentally during naturalistic stimulation (S9A Fig). (D) Information gain rate as function of mean noise correlation for heterogenous (ρsignal = 0) and homogenous (green) conditions for mean signal correlation ρsignal = 0.6. For heterogeneous conditions, we compared values obtained when signal and noise correlations were independent of one another (purple) to those obtained when signal and noise correlations were related as seen experimentally during naturalistic stimulation (orange). Error bands indicate 1 SEM (N = 40 simulations). It is seen that information rates were lower when signal and noise correlations were related, consistent with previous studies, but were still higher than those obtained in the homogeneous condition. (TIF) [file pbio.3002623.s010.TIF]

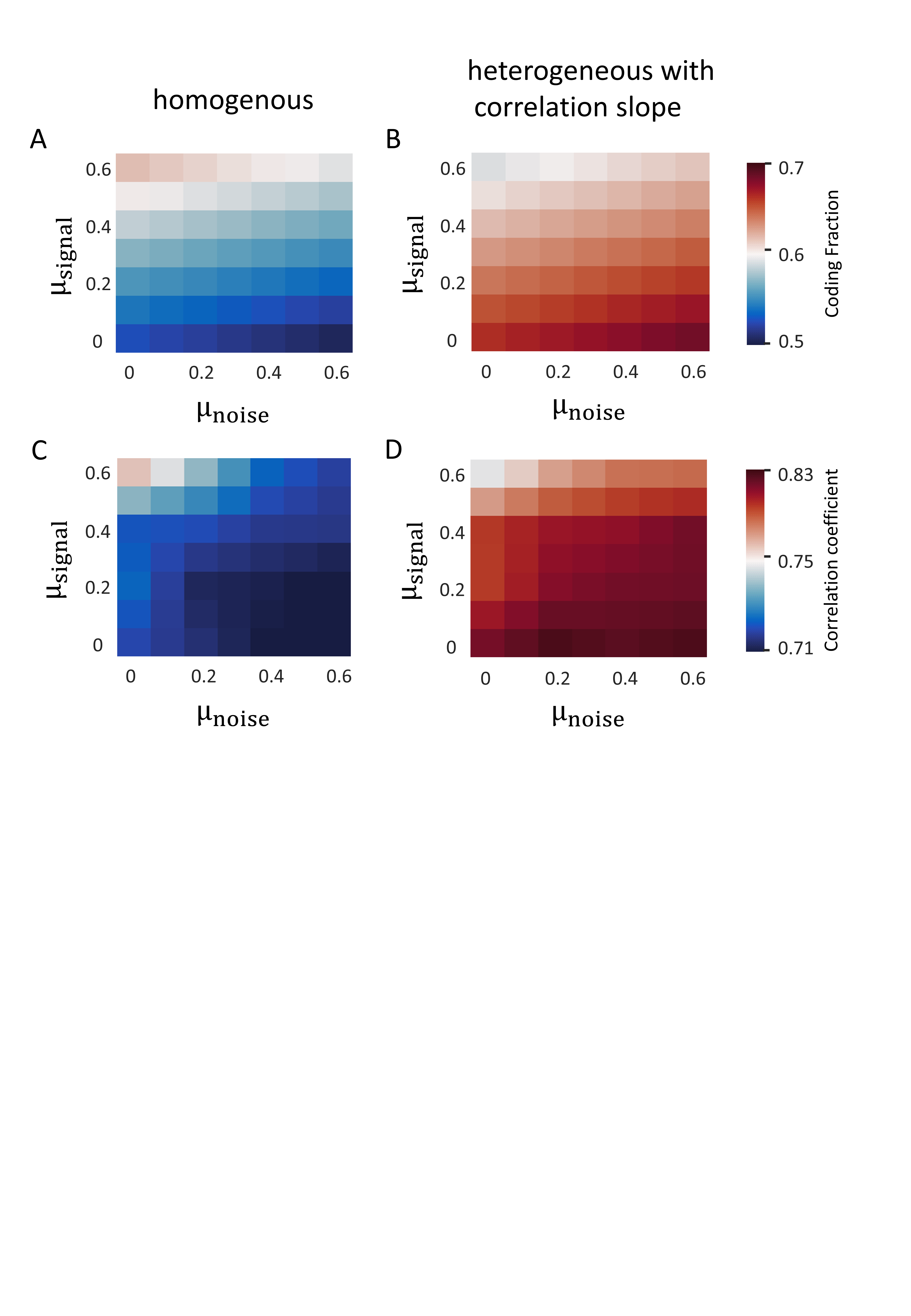

Supplement: S11 Fig — (A) Coding fraction as a function of mean signal and mean noise correlations for the homogeneous case. For given values of mean signal and noise correlations, we assumed that signal and noise correlations were independent of one another as seen experimentally during artificial stimulation (S9B Fig). (B) Coding fraction as a function of mean signal and mean noise correlations for the heterogeneous case where the relationship between signal and noise correlations has the same slope as that seen experimentally during naturalistic stimulation (S9A Fig). (C) Correlation coefficients as a function of mean signal and noise correlations for the homogenous conditions. As for the simulations in panel A, we assumed that signal and noise correlations were independent as seen experimentally. (D) Correlation coefficients as a function of mean signal and noise correlations for the heterogenous conditions. As for the simulations in panel B, we assumed that signal and noise correlations were correlated with the same slope as seen experimentally. As seen for the information rate (S10A Fig), coding fraction and correlation coefficient values increase with higher signal correlations and lower noise correlations when during homogenous condition. In contrast, as seen for the information rate (S10C Fig), coding fraction and correlation coefficient values increase with higher noise correlations and lower signal correlations when during heterogenous population activity. (TIF) [file pbio.3002623.s011.TIF]

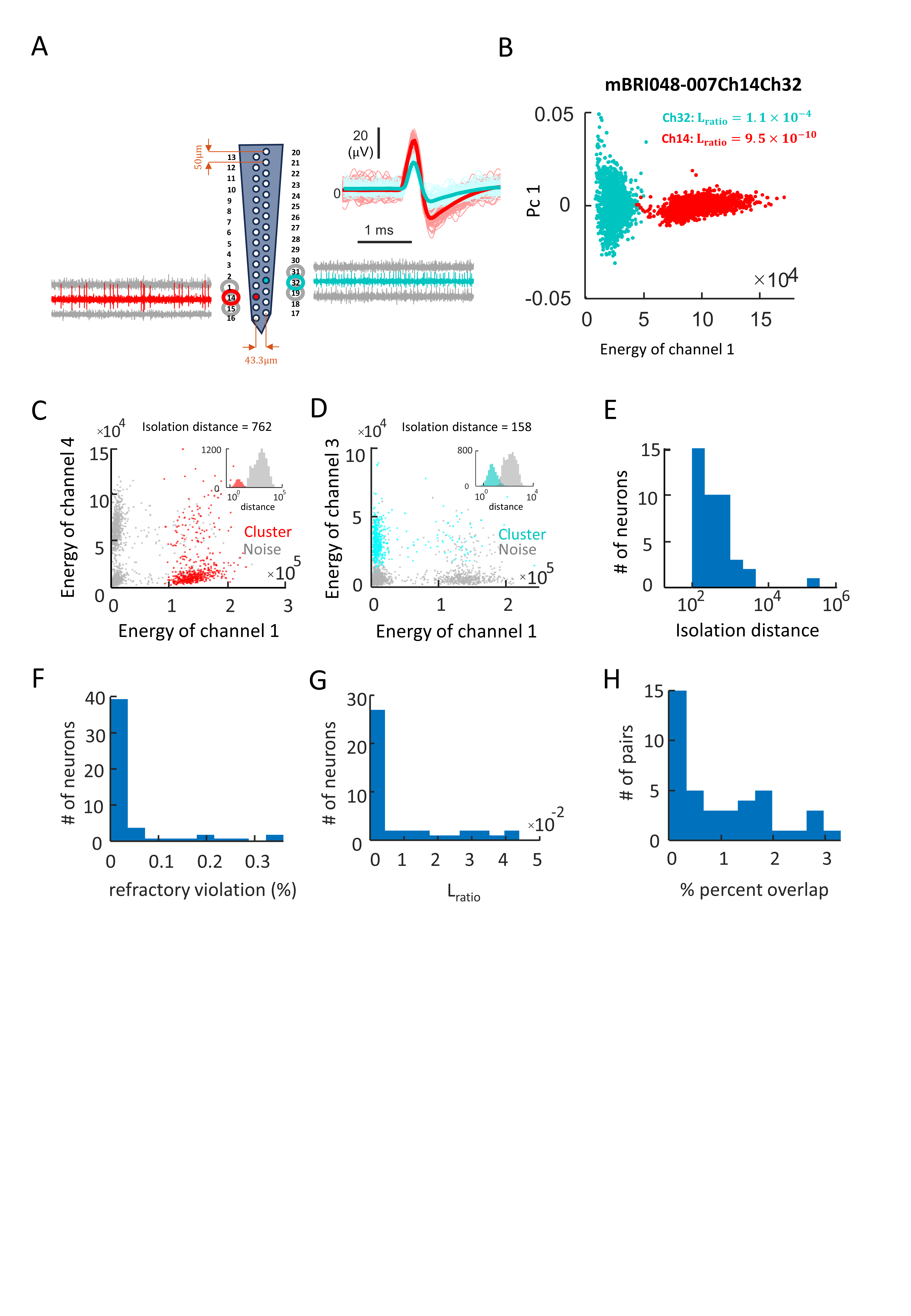

Supplement: S12 Fig — (A) Schematic of the multi-channel probe and signals recorded on separate sites (14 in red and 32 in cyan, together with adjacent sites; schematic reproduced from manual for V1x32-Poly2-15mm-50s-177 manual from NeuroNexus website, https://neuronexus.com/). Also shown are extracellular spike waveforms aligned to time of occurrence (thin curves) and population averages (thick curves). (B) Plot in feature space of both units. The features in x-axis and y-axis are the energy of spikes and first principal component of feature matrix, respectively, as describe in methods section. Red and blue dots represent scatter plots for spikes of first and second units obtained from channels 14 and 24 on the probe, respectively. The L-ratio values for each neuron are indicated in the figure. (C) Scatter plot of spike clusters of the first unit versus noise in feature space. Energy of spikes in channels 1 and 4 are used for this plot. The isolation distance computed between cluster and noise spikes is 762 for this unit. (D) Scatter plot of spike clusters of the second unit versus noise in feature space. Energy of spikes in channels 1 and 3 are used for this plot. The isolation distance computed between cluster and noise spikes is 158 for this unit. In both panels C and D, a third of spikes are shown randomly for visualization purposes. (E) Histogram of isolation distance calculated for all units (N = 41). All values were above 100 indicating good if not excellent separation between cluster and noise spikes. (F) Histogram of refractory violation for single neurons in the population (N = 41). (G) Lratio calculated for all the neurons in the population (N = 41). All values are less than 0.05. (H) Percentage overlap between clusters of neurons in pairs (similar to that in panel B; N = 35). (TIF) [file pbio.3002623.s012.tif]
